# Stunting Status and Exposure to Infection and Inflammation in Early Life Shape Antibacterial Immune Cell Function Among Zimbabwean Children

**DOI:** 10.3389/fimmu.2022.899296

**Published:** 2022-06-13

**Authors:** Kuda Mutasa, Joice Tome, Sandra Rukobo, Margaret Govha, Patience Mushayanembwa, Farai S. Matimba, Courage K. Chiorera, Florence D. Majo, Naume V. Tavengwa, Batsirai Mutasa, Bernard Chasekwa, Jean H. Humphrey, Robert Ntozini, Andrew J. Prendergast, Claire D. Bourke

**Affiliations:** ^1^ Zvitambo Institute for Maternal and Child Health Research, Harare, Zimbabwe; ^2^ Department of International Health, Johns Hopkins Bloomberg School of Public Health, Baltimore, MD, United States; ^3^ Centre for Genomics and Child Health, Queen Mary University of London, London, United Kingdom

**Keywords:** Immune Function, Zimbabwe, Malnutrition, Stunting, Inflammation, Maternal and Child Health, Pregnancy, Immune Cells

## Abstract

**Background:**

Children who are stunted (length-for-age Z-score<-2) are at greater risk of infectious morbidity and mortality. Previous studies suggest that stunted children have elevated inflammatory biomarkers, but no studies have characterised their capacity to respond to new infections (i.e., their immune function). We hypothesised that antibacterial immune function would differ between stunted and non-stunted children and relate to their health and environment during early life.

**Methods:**

We enrolled a cross-sectional cohort of 113 HIV-negative children nested within a longitudinal cluster-randomised controlled trial of household-level infant and young child feeding (IYCF) and water, sanitation and hygiene (WASH) interventions in rural Zimbabwe (SHINE; Clinical trials registration: NCT01824940). Venous blood was collected at 18 months of age and cultured for 24 h without antigen or with bacterial antigens: heat-killed *Salmonella typhimurium* (HKST) or *Escherichia coli* lipopolysaccharide (LPS). TNFα, IL-6, IL-8, IL-12p70, hepcidin, soluble (s)CD163, myeloperoxidase (MPO) and IFNβ were quantified in culture supernatants by ELISA to determine antigen-specific immune function. The effect of stunting status and early-life exposures (anthropometry, inflammation at 18 months, maternal health during pregnancy, household WASH) on immune function was tested in logit and censored log-normal (tobit) regression models.

**Results:**

Children who were stunted (n = 44) had higher proportions (86.4% vs. 65.2%; 88.6% *vs*. 73.4%) and concentrations of LPS-specific IL-6 (geometric mean difference (95% CI): 3.46 pg/mL (1.09, 10.80), p = 0.035) and IL-8 (3.52 pg/mL (1.20, 10.38), p = 0.022) than non-stunted children (n = 69). Bacterial antigen-specific pro-inflammatory cytokine concentrations were associated with biomarkers of child enteropathy at 18 months and biomarkers of systemic inflammation and enteropathy in their mothers during pregnancy. Children exposed to the WASH intervention (n = 33) produced higher LPS- (GMD (95% CI): 10.48 pg/mL (1.84, 60.31), p = 0.008) and HKST-specific MPO (5.10 pg/mL (1.77, 14.88), p = 0.003) than children in the no WASH group (n = 80). There was no difference in antigen-specific immune function between the IYCF (n = 55) and no IYCF groups (n = 58).

**Conclusions:**

Antibacterial immune function among 18-month-old children in a low-income setting was shaped by their stunting status and prior exposure to maternal inflammation and household WASH. Heterogeneity in immune function due to adverse exposures in early life could plausibly contribute to infection susceptibility.

## Introduction

The first 1,000 days of life (between conception and a child’s second birthday) is a critical window for the development of long-term immune defence against infections (1). Much of the variation between individual immune phenotype and function is known to be non-heritable (2, 3); a range of drivers including infections, maternal health status, microbiome, urbanisation, vaccinations, environmental contaminants (e.g., cigarette smoke) and diet are implicated for healthy children and adults (4, 5). However, empirical studies of how cellular immune function develops in young children are limited in low- to- middle-income countries (LMIC) (1, 6, 7), where the health burden of infections is aggregated (8).

Stunting (height- or length-for-age Z score (LAZ) < -2) is an indicator of chronic undernutrition, can originate *in utero* and affects 144 million children under 5 years old, predominantly in LMIC (8). Stunted children have high symptomatic and asymptomatic pathogen carriage (9) and are at greater risk of infectious mortality than their non-stunted peers (10). However, linear growth deficits are more likely an indicator than a cause of infectious susceptibility among undernourished children (11). There is some evidence that systemic inflammatory mediators are consistently higher (e.g., 6 weeks to 6 months (12), birth to 12 months (13)) in stunted versus non-stunted children, positively associated with circulating pathogen-associated molecular patterns (PAMPs) and anti-pathogen antibodies (14) but negatively associated with linear growth factors (12, 13, 15). Environmental enteropathy, a condition which develops in the context of asymptomatic enteropathogen carriage, recurrent infection, intestinal damage and inflammation, is thought to underlie the relationship between stunting and systemic inflammation (9, 16), primarily *via* translocation of PAMP from microbes in the gut into circulation (9, 14). For example, concentrations of lipopolysaccharide (LPS, a component of gram-negative bacteria also termed endotoxin) in plasma samples from a cohort of infants in rural Gambia were found to be twice the upper reference limit for infants in high-income settings and negatively associated with their linear and ponderal growth (14). However, no studies to date have directly assessed whether immune cells from stunted children respond differently to PAMP relative to those of non-stunted children, how these functional differences originate or whether they relate to subsequent infection risk and severity.

The goal of this study was to assess blood immune cell function among 18-month-old children in rural Zimbabwe, which has an estimated 23.5% prevalence of stunting among children under 5 years old (8), high rates of adverse birth outcomes (17) and endemic pathogen exposure (18, 19). We focused on PAMPs derived from two gram-negative bacteria commonly isolated from stool samples collected from children in LMIC (purified LPS from *Escherichia coli* and whole heat-killed *Salmonella typhimurium*, HKST) (15, 16) and soluble mediators typically upregulated upon initial PAMP recognition by surface-expressed toll-like receptors (TLR) on innate immune cells. We compared bacterial antigen-specific immune-mediator secretion between children who were stunted versus non-stunted and used longitudinal data on each child, their mother and household environment from pregnancy through to 18 months to identify early-life factors associated with immune heterogeneity. Our data provide a holistic view of the capacity of immune cells to respond to a new bacterial challenge in a cohort of young children exposed to a range of adverse environmental exposures in early life.

## Materials and Methods

### Study Ethics

This is a sub-study of the Sanitation Hygiene and Infant Nutrition Efficacy trial (SHINE (20); ClinicalTrials.gov, number NCT01824940), a cluster-randomised 2 × 2 factorial trial of the impact of household water, sanitation and hygiene (WASH) and/or infant and young child feeding (IYCF) interventions on linear growth and haemoglobin among children in rural Zimbabwe (19, 21). Ethical approval for SHINE was provided by the Medical Research Council of Zimbabwe and the Institutional Review Board of the Johns Hopkins Bloomberg School of Public Health. All participants provided written informed consent for themselves and their children to participate.

### Study Participants

Participants were pregnant women who were permanent rural residents of Chirumanzu or Shurugwi district, Zimbabwe; became pregnant during SHINE enrolment (November 2012-March 2015); and consented to participation during pregnancy. Women were recruited throughout their pregnancies. Prevalence of maternal HIV and urogenital schistosomiasis, adverse birth outcomes and child stunting are high in these communities; however, malaria prevalence is low (17–19). Additional inclusion criteria for this sub-study were as follows: children born to SHINE mothers who completed an 18-month study visit between July 2016 and July 2017; provided a blood sample ≥2 mL to allow for all whole-blood culture assays; and lived in the catchment area of Shurugwi District Hospital or St. Theresa’s Hospital, where trial hubs with laboratory facilities for cell culture were based.

### SHINE Interventions

SHINE was cluster-randomised by the catchment area of one to four Village Health Workers (VHW) working for Zimbabwe’s Ministry of Health and Child Care. VHWs made 15 home visits to SHINE participants at approximately monthly intervals between enrolment and 12 months after birth. VHWs delivered interactive behaviour-change interventions within one of four arms: standard of care (SOC): promoted breastfeeding (early initiation, exclusive to 6 months and prolonged), family planning, prevention-of-mother-to-child transmission of HIV services and childhood immunisations; WASH: SOC interventions plus households received a Blair ventilated improved pit latrine, two handwashing stations, water chlorination (WaterGuard, Nelspot, Zimbabwe) and a play space to separate infants from animal faeces; IYCF: SOC interventions plus promoted nutrient-dense, diverse infant diets and provided small-quantity lipid-based nutrient supplementation (SQ-LNS, 20 g/day) for infants from 6 to 18 months; and combined WASH + IYCF: all SOC, WASH and IYCF interventions. Fidelity of intervention delivery was high for all intervention arms: >98% of WASH households received ventilated improved pit latrines and handwashing stations, 92% received play mats and play yards; 80% received ≥80% of the planned deliveries of soap and chlorine solution; and 79% of IYCF households received ≥80% of planned deliveries of SQ-LNS (21). This sub-study recruited children across the four trial arms.

Research nurses visited households at baseline (approximately 2 weeks after enrolment), 32 weeks’ gestation and 1, 3, 6, 12 and 18 months after birth. Maternal anthropometry (height, weight, mid-upper-arm circumference (MUAC)), haemoglobin (HemoCue, Ängelholm, Sweden), *Schistosoma haematobium* (urinary microscopy), and HIV status (*via* the Zimbabwe National HIV testing algorithm (22)) were measured, blood and stool samples collected and maternal and household characteristics assessed by questionnaire at baseline. Birthweight (Tanita scales; Amsterdam, Netherlands) and delivery characteristics were recorded at birth. Child anthropometry and symptoms of infection (7-day caregiver recall questionnaire) and blood and stool sampling were done at all postnatal visits. At the 18-month visit, child haemoglobin was measured by HemoCue and median length calculated from three measurements.

### Plasma and Stool ELISA

Biomarkers of systemic inflammation (capsular polysaccharide-reactive protein (CRP), sCD14) and environmental enteropathy (intestinal fatty acid-binding protein (IFABP)) were quantified in plasma by enzyme-linked immunosorbent assay (ELISA) (23). Biomarkers of intestinal inflammation and environmental enteropathy (myeloperoxidase (MPO), neopterin, α-1-antitrypsin (AAT)) were measured by stool ELISA (23).

### Whole Blood Cultures

Blood was collected from each child by venipuncture at their 18-month visit. Six hundred millilitres of blood was diluted in RPMI 1640 medium supplemented with 1% v/v penicillin–streptomycin (GIBCO, Amarillo, USA) and cultured in 24-well plates for 24 h under three parallel conditions (one culture well/condition; culture conditions for the study were pre-prepared and stored at -80°C until use to minimise inter-plate variation): without stimulus (Media); 0.25 EU/mL ultrapure lipopolysaccharide from *E. coli* (LPS); and 1 × 10^8^ cells/mL heat-killed *Salmonella enterica* serovar Typhimurium (HKST; Invivogen, Toulouse, France). Cultures were maintained at 37°C, 6% CO_2_, using the CO2Gen Compact system (Oxoid, Basingstoke, UK). Cell-free culture supernatants were harvested and stored at -80°C. Supernatants were transported to Zvitambo Institute laboratory, Harare, where TNFα, IL-6, IL-8, IL-12p70, hepcidin, sCD163, MPO (Bio-Techne DuoSet, Abingdon, UK) and IFNβ (PBL VeriKine, Piscataway, USA) concentrations were quantified in duplicate ELISA relative to eight-point standard curves. Mediators were chosen to reflect key domains of innate immune cell function: pro-inflammatory cytokines (TNFα and IL-6) that trigger the liver acute-phase response; IFNβ known for its antiviral and antibacterial action (24); IL-8, a pro-inflammatory cytokine and neutrophil chemoattractant; sCD163, a bacterial sensor shed by activated monocytes/macrophages (25); IL-12p70 which promotes antibacterial/antiviral T-cell function; hepcidin which inhibits cellular iron efflux to regulate iron homeostasis and/or defend against extracellular pathogens (26); and MPO, an enzyme which catalyses bactericidal reactive oxygen species (ROS) production (27). ELISA lower limits of detection were as follows: TNFα—15.6 pg/mL, IL-6—4 pg/mL, IL-8—31.2 pg/mL, IL-12p70—31.2 pg/mL, sCD163—156 pg/ml, hepcidin—3.1 pg/mL, MPO—62.5 pg/mL and IFNβ—50 pg/mL. Concentrations below the assay detection limit were censored at the limit of detection; those greater than the top standard concentration were rerun at higher dilution and multiplied by the dilution factor.

### Statistical Analysis

A sample size calculation based on previous estimates was not done as there are no existing comparisons of immune function variables by stunting. The sub-study analysis plan was published prior to data analysis (https://osf.io/rfhve/). SHINE intervention arm analyses were by intention to treat.

Sub-study cohort characteristics were compared to the SHINE cohort and between SHINE arms by generalised estimating equations (means), Somers’ D (medians) and logit regression (proportions).

Log-transformed mediator concentrations were compared between culture conditions using generalised estimating equations to identify mediators for which there was a detectable increase in response to bacterial antigen stimulation above unstimulated cultures. For mediators with a detectable antibacterial response, concentrations in unstimulated culture supernatants were subtracted from those in LPS- and HKST-stimulated culture supernatants from the same child to give antigen-specific mediator concentrations (Δ); antigen-specific concentrations < unstimulated were censored at the mean of the lowest detectable value for the cohort/2. Proportions of children with unstimulated concentrations > ELISA detection limit and proportions of children with antigen-specific concentrations > unstimulated levels were compared between groups by unadjusted logit regression; odds ratios (OR) and 95% confidence intervals (95% CI) are reported.

Censored antigen-specific mediator concentrations were not normally distributed; therefore, censored log-normal (tobit) regression was used to assess the effects of child, maternal and SHINE intervention variables on these outcome measures. We developed separate causal inference models to assess the total effect of four groups of exposure variables (18-minth stunting status; 18-month inflammation; baseline maternal health; SHINE intervention arm) on immune function outcome variables (i.e., antigen-specific immune-mediator concentrations), adjusting for confounders (**Supplementary Table 1**). Confounder variables were selected for inclusion in each of the adjusted models from a full list of variables available from the SHINE study (**Supplementary Table 2**) using directed acyclic graphs (DAG) based on existing research literature. Variables with ≥20% missingness and/or ≤10 total events (continuous variables) or events per category (categorical variables) were not included in regression models. The SHINE intervention arm and hub were included in all adjusted models where the exposure was measured after the SHINE baseline visit. We estimated the geometric mean difference (GMD) in immune mediators (pg/mL) by exponentiating unadjusted and adjusted censored log-normal (tobit) regression coefficients and associated 95% CI; results are reported as GMD between case/intervention group and control/no intervention group for categorical exposure variables and geometric mean increase per one-unit increase in exposure for continuous exposure variables.

Sensitivity analyses assessed the modifying effects of exposure to maternal HIV, 18-month anaemia (haemoglobin <10.5 g/dL; predefined per SHINE secondary outcome (20)) and presence of any symptom of infection in the week preceding the 18-month visit on the relationship between immune function and stunting status.

Statistical analyses were performed using Stata version 14 (StataCorp, College Station, TX) and Prism version 9 (GraphPad Software, La Jolla, CA).

## Results

### Characteristics of the Study Cohort

**Figure 1:**
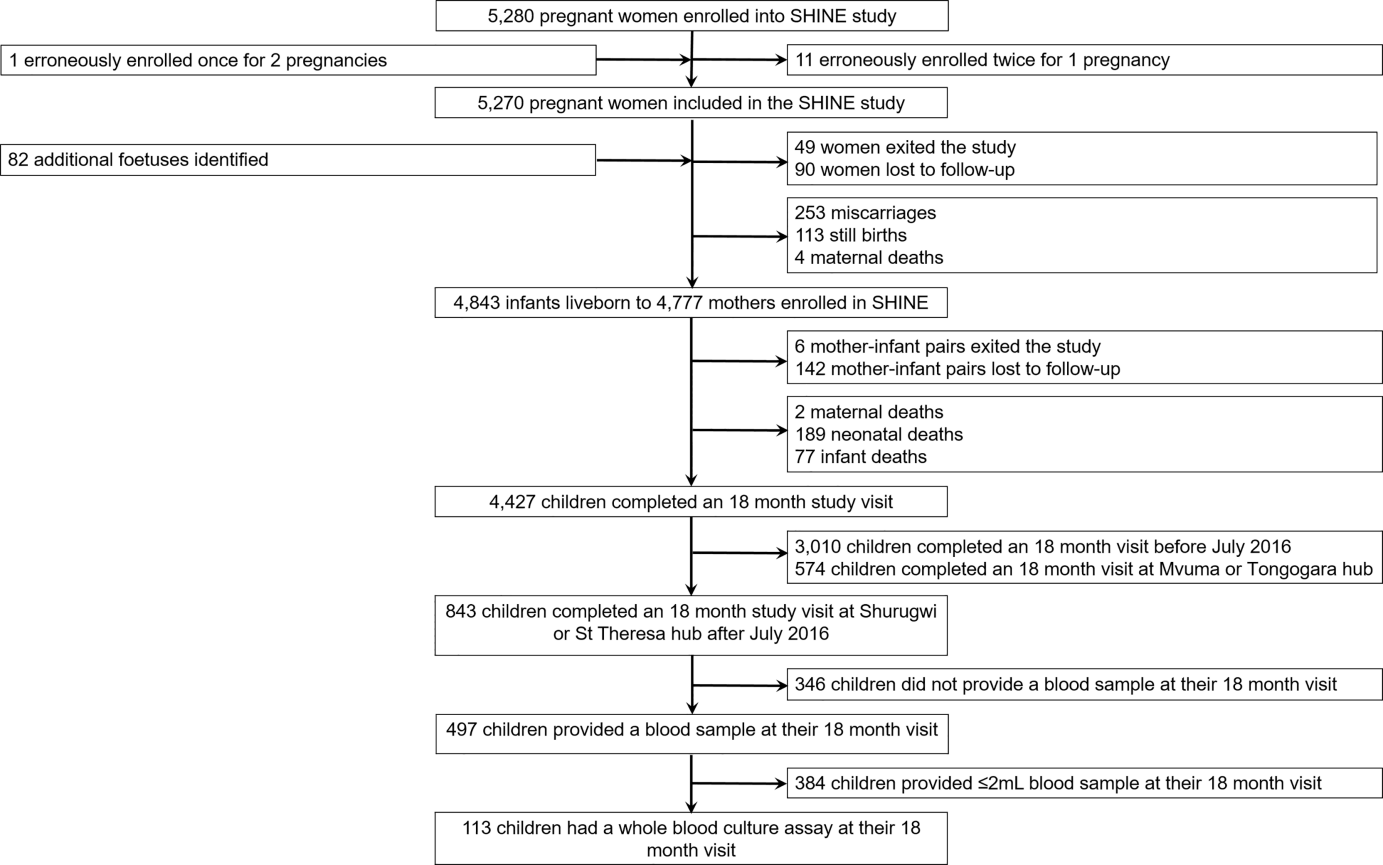
Selection of 18-month-old infants for inclusion in the immune function sub-study of SHINE.

One hundred children (53.1% female; mean age: 18.4 months, standard deviation: 1.6) born to 111 mothers were included in the immune function sub-study of SHINE (**Figure 1**; **Table 1**). Characteristics of the children enrolled in the sub-study were broadly comparable to those of the main SHINE trial cohort although the sub-study cohort tended to be healthier, including more institutional deliveries, term and appropriate-for-gestational age births and fewer preterm births and caregiver-reported symptoms of infection (**Supplementary Table 2**). Six children (5.4%) were born low birthweight (<2.5 kg). All sub-study participants were HIV-negative, but 18 (16.0%) were HIV-exposed, uninfected (HEU). At the 18-month visit, 12 children (10.6%) were anaemic (haemoglobin <10.5 g/dL). Of the 11 (9.7%) children for whom symptoms of infection were reported in the previous 7 days, two had diarrhoea, five had fever and seven had a cough.

**Table 1:** Characteristics of infants enrolled in the SHINE immune function sub-study.

Characteristics	Enrolled in the SHINE immune function sub-study
**Infants, N**	113
**SHINE hub:**	
Shurugwi District Hospital, n/N (%)	81/113 (71.7%)
St. Theresa’s Hospital, n/N (%)	32/113 (28.3%)
**SHINE intervention arms:**	
Standard of care (SOC), n/N (%)	42/113 (37.2%)
Infant and young child feeding (IYCF), n/N (%)	38/113 (33.6%)
Water, sanitation and hygiene (WASH), n/N (%)	16/113 (14.2%)
IYCF & WASH, n/N (%)	17/113 (15.0%)
**At birth:**	
Female sex, n/N (%)	60/113 (53.1%)
Birthweight, mean (SD) [n], kg	3.2 (0.4) [111]
Low birthweight <2.5 kg, n/N (%)	6/111 (5.4%)
Birth outcome, n/N (%):	
Term, Appropriate for gestational age (AGA)	45/49 (91.8%)
Preterm, AGA	4/49 (8.2%)
Preterm, Small for gestational age (SGA)	0/8 (0.0%)
**At 18-month visit:**	
Age, n (mean) (SD), months	18.4 (1.6) [113]
Anthropometry:	
Length-for-age Z score (LAZ), mean (SD) [n]	-1.6 (1.1) [113]
Weight-for-height Z score (WHZ), mean (SD) [n]	0.02 (1.1) [113]
Weight-for-age Z score (WAZ), mean (SD) [n]	-0.7 (1.1) [113]
MUAC-for-age Z score (MUACZ), mean (SD) [n]	0.16 (0.9) [113]
Head circumference-for-age Z score (HCZ), mean (SD) [n]	-0.1 (1.1) [113]
Stunted (LAZ<-2)	44/113 (38.9%)
Anaemic (haemoglobin <10.5 g/dL), n/N (%)	12/113 (10.6%)
Caregiver reported symptoms (7-day recall questionnaire), n/N (%):	
Any symptom (≥1 of the following symptoms)	11/113 (9.7%)
Diarrhoea	2/113 (1.8%)
Acute respiratory infection	0/96 (0.0%)
Fever	5/96 (5.2%)
Cough	7/95 (7.4%)
Pus from ear	0/95 (0.0%)
Difficulty feeding	0/95 (0.0%)
Blood in stool	0/95 (0.0%)
Mucus in stool	0/95 (0.0%)
Systemic inflammation:	
Plasma sCD14, median (IQR) [n], pg/mL	1,387,951 (1,082,782; 1,702,291) [82]
Plasma CRP, median (IQR) [n], ng/mL	1,314 (42; 5,957) [82]
Intestinal inflammation:	
Stool MPO, median (IQR) [n], ng/mL	1,887 (1,070; 4,118) [72]
Stool neopterin, median (IQR) [n], nmol/L	356.1 (109; 785) [72]
Biomarkers of gut damage:	
Plasma intestinal fatty acid-binding protein (IFABP), median (IQR) [n], pg/mL	1,230 (842; 1,659) [82]
Stool α-1-antitrypsin (AAT), median (IQR) [n], ng/mL	314,787 (142,167; 588,501) [72]
HIV status, n/N (%):	
HIV-unexposed uninfected (HUU)	95/113 (84.1%)
HIV-exposed uninfected (HEU)	18/113 (15.9%)
HIV positive	0/113 (0.0%)
HIV unknown	0/113 (0.0%)
ART initiated for HEU, n/N (%)	16/16 (100.0%)
Cotrimoxazole initiated for HEU, n/N (%)	12/13 (92.3%)

### Blood Immune Cell Mediator Secretion in Response to Bacterial Antigens

**Figure 2:**
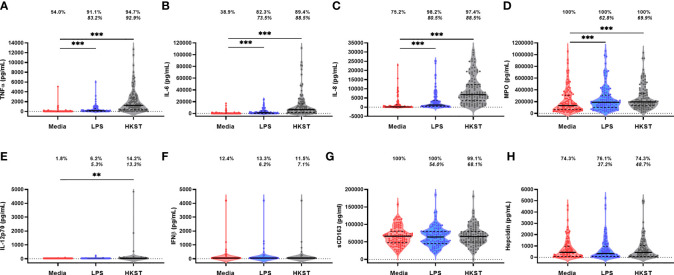
Soluble mediator production by blood immune cells from 18-month-old Zimbabwean children in response to *in vitro* bacterial antigen challenge. Violin plots (median and interquartile range indicated) of **(A)** TNFα; **(B)** IL-6; **(C)** IL-8; **(D)** MPO; **(E)** IL-12p70; **(F)** IFNβ; **(G)** sCD163; and **(H)** hepcidin concentrations (pg/mL) present in supernatants from parallel 24h culture of whole blood samples from Zimbabwean children with culture media only (unstimulated; red), LPS (blue) and HKST (grey). Proportions indicate participants with mediator concentration > ELISA limit of detection; proportions in italics indicate participants with mediator concentration > unstimulated. Log-transformed mediator concentrations were compared between culture conditions using generalised estimating equations (n = 113); **p < 0.01, ***p < 0.001.

Immune-mediator concentrations and proportions of the cohort producing detectable unstimulated and LPS- and HKST-stimulated mediators in 24h whole-blood cultures are shown in **Figure 2**. Mediator responses to purified LPS are indicative of cellular activation *via* TLR4 whereas HKST responses reflect activation *via* a combination of receptors, primarily TLR2 and TLR4. Cytokines (TNFα, IL-6, IL-8, IL-12p70 and IFNβ) tended to be present at low/undetectable levels in unstimulated cultures, but most participants had detectable MPO, sCD163 and hepcidin in unstimulated cultures, indicative of non-specific mediator production and/or pre-culture plasma concentrations (**Figures 2D, G, H**). There was evidence that TNFα, IL-6, IL-8 and MPO concentrations were higher in LPS- and HKST-stimulated versus unstimulated cultures (**Figures 2A–D**), consistent with activation of bacterial antigen-specific responses. Concentrations of IL-12p70 were also higher in HKST-stimulated versus unstimulated cultures despite only 14.2% of children having detectable IL-12p70 (**Figure 2E**). There was limited evidence for higher production of sCD163, hepcidin or IFNβ in bacterial antigen-stimulated cultures and of IL-12p70 in LPS-stimulated cultures versus unstimulated cultures (**Figures 2E–H**), suggesting that blood immune cells from participants did not upregulate these mediators upon bacterial antigen challenge *in vitro*. Based on these data, we went on to explore heterogeneity in antibacterial immune function using ΔTNFα, ΔIL-6, ΔIL-8 and ΔMPO between antigen-stimulated and unstimulated cultures (i.e., antigen-specific mediator levels).

### Immune Function of Stunted Versus Non-Stunted Children at 18 Months

**Figure 3:**
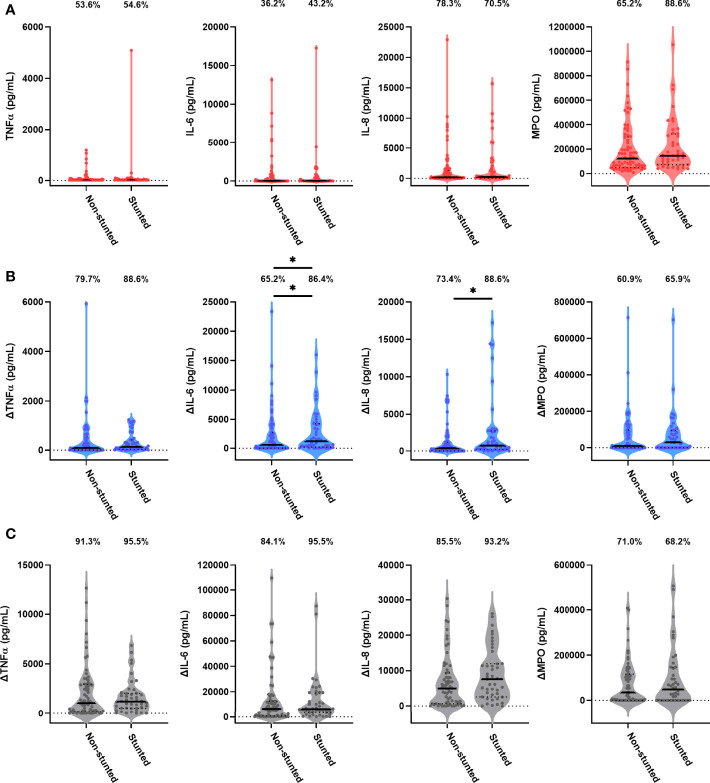
Unstimulated and bacterial antigen-stimulated immune-mediator production by blood immune cells from stunted versus non-stunted children. Violin plots (median and interquartile range indicated) of **(A)** unstimulated (red), **(B)** LPS-stimulated (blue) and **(C)** HKST-stimulated (grey) TNFα, IL-6, IL-8 and MPO concentrations in 24h culture supernatants. LPS- and HKST-specific concentrations (Δ) were calculated for each child by subtracting concentrations present in matched unstimulated culture supernatants. Proportions indicate participants with mediator concentration > ELISA limit of detection **(A)** and participants with antigen-stimulated mediator concentration > unstimulated **(B, C)**. Proportions of children with detectable mediator levels and mediator concentrations were compared by stunting status (stunted n = 44 versus non-stunted n = 69) *via* multinomial logit regression and censored log-normal (tobit) regression, respectively (unadjusted analyses indicated; full results in **Table 2**); *p < 0.05.

Forty-four children (38.9%) were stunted at the 18-month visit consistent with prevalence of stunting in the SHINE cohort (33.2%; **Supplementary Table 2**). We hypothesised that stunting status is associated with antibacterial immune function. In causal inference models, we adjusted for the SHINE arm and hub, infant sex due to its impact on immune development (28) and because boys had poorer linear growth than girls in SHINE (21) and birthweight, which was associated with both 18-month stunting and immune function. The stunted and non-stunted groups had comparable proportions of children with detectable TNFα, IL-6, IL-8 and MPO in unstimulated cultures (**Figure 3A**). Comparisons of other unstimulated mediator proportions and concentrations by stunting status are provided in **Supplementary Figure 1** and **Supplementary Table 3**.

Higher proportions of children who were stunted versus non-stunted at 18 months had detectable LPS- and HKST-specific mediators, and concentrations of all antigen-specific mediators were higher in stunted versus non-stunted children (**Figures 3B, C**). This was most evident for proportions of children producing detectable LPS-specific IL-6 (logit regression OR: 3.38, 95% CI: 1.17, 9.75; p = 0.024) and for concentrations of LPS-specific IL-6 and LPS-specific IL-8 in unadjusted models (**Figure 3B**; **Table 2**). The stunted group produced 3.46 pg/mL (95% CI: 1.09, 10.80; p = 0.035) more IL-6 and 3.52 pg/mL (95% CI: 1.20, 10.38; p = 0.022) more IL-8 in response to LPS than the non-stunted group. The magnitude of the difference in LPS-specific IL-6 and IL-8 concentrations between stunted and non-stunted children was reduced in adjusted models (**Table 2**). These data are the first evidence that stunted children have a distinct TLR4-mediated response to LPS compared to non-stunted children; we did not find evidence for a difference in responses to HKST which immune cells detect *via* multiple receptors.

**Table 2:** Censored log-normal (tobit) regression analysis of the relationship between child stunting status and immune-mediator production at 18 months of age.

Outcome	Unadjusted			Adjusted for SHINE arm, hub, sex, and birthweight		
	GMD*^a^*	95% CI	p	Adj. GMD*^a^*	Adj. 95% CI	p
**Unstimulated** * ^b^ *:						
TNFα	0.84	0.41; 1.72	0.622	0.97	0.46; 2.03	0.932
IL-6	1.63	0.14; 19.7	0.696	3.06	0.24; 38.85	0.385
IL-8	0.91	0.35; 2.34	0.847	1.36	0.50; 3.71	0.547
MPO	1.30	0.90; 1.86	0.164	1.22	0.86; 1.75	0.262
**Antigen-specific** * ^c^ *:						
LPS-specific TNFα	3.19	0.71; 14.29	0.130	2.16	0.45; 0.42	0.334
HKST-specific TNFα	1.30	1.68; 2.83	0.510	0.85	0.42; 1.72	0.659
LPS-specific IL-6	3.46	1.09; 10.80	0.035	2.07	0.71; 6.05	0.181
HKST-specific IL-6	1.73	0.74; 4.01	0.203	1.03	0.51; 2.10	0.923
LPS-specific IL-8	3.52	1.20; 10.38	0.022	2.83	0.90; 8.76	0.073
HKST-specific IL-8	2.16	0.92; 5.05	0.075	1.75	0.78; 3.90	0.173
LPS-specific MPO	1.67	0.31; 9.02	0.554	0.70	0.12; 4.05	0.696
HKST-specific MPO	1.12	0.41; 3.03	0.823	0.70	0.25; 1.90	0.482

^a^Geometric mean difference in mediator concentration (pg/mL) between the stunted group (n = 44) and the non-stunted group (n = 69) estimated from censored log-normal (tobit) regression coefficient; bolded text indicates mediators with evidence for an association with 18-month stunting status (p < 0.05).

^b^Concentrations in unstimulated whole blood culture supernatants.

^c^ΔConcentrations between antigen-stimulated and unstimulated whole blood culture supernatants.

Due to the low number of children who were HIV-exposed uninfected, anaemic and/or had symptoms of infection at 18 months of age, we investigated the modifying effects of these characteristics on the association between immune function and stunting status in sensitivity analyses (**Supplementary Figures 2, 3**). We found evidence that GMD in LPS-specific IL-6 and IL-8 between stunted and non-stunted children was higher when HIV-exposed (GMD (95% CI): ΔIL-6: 3.56 (1.15, 11.1), p = 0.028; ΔIL-8: 4.62 (1.46, 14.73), p = 0.035) and anaemic children (ΔIL-6: 4.62 (1.46, 14.73); p = 0.009; ΔIL-8: 4.18 (1.32, 13.33), p = 0.015) were excluded, but lower when symptomatic children were excluded (ΔIL-6: 3.13 (0.95, 10.28), p = 0.061; ΔIL-8: 3.06 (1.12, 9.30), p = 0.048). These data support the hypothesis that HIV status, anaemia and infectious symptoms modify how stunted children respond to bacterial antigens in the small number of affected children in our cohort; future studies powered to compare these clinical groups are required.

### Relationship Between Anthropometry and Antibacterial Immune Function at 18 Months

Given that stunting is characterised clinically as low height-for-age, we investigated whether immune function was associated with age-adjusted measures of anthropometry across both the stunted and non-stunted groups. There was limited evidence for a continuous relationship between any bacterial antigen-specific mediators and 18-month anthropometry (LAZ, weight-for-height, weight-for-age, MUAC or head circumference Z scores) before or after adjustment for the SHINE arm, hub, infant sex and maternal HIV status (p > 0.05; data not shown). However, antigen-specific MPO concentrations increased per 100-g increase in birthweight across the cohort (n = 111; GMD (95% CI) LPS: 0.14 (0.02, 1.03), p = 0.053; HKST: 0.29 (0.09, 0.93), p = 0.037); there was no evidence for an association between birthweight and antigen-specific TNFα, IL-6 or IL-8 (**Supplementary Figure 4**). We did not find evidence for an association between LAZ at 3 (n = 92), 6 (n = 95) or 12 months (n = 91) and any antigen-specific immune mediators (p > 0.05; data not shown). Thus, differences in LPS-specific IL-6 and IL-8 between stunted and non-stunted children were not solely explained by a continuous relationship between immune function and children’s current or preceding anthropometry. We went on to explore alternative, non-anthropometric characteristics of stunting that could contribute to immune heterogeneity in this cohort.

### Relationship Between Inflammation and Antibacterial Immune Function at 18 Months

Biomarkers of systemic inflammation (plasma CRP, sCD14) and intestinal inflammation (stool MPO and neopterin) are non-specific indicators of immune cell activation, which can be elevated as a result of infection and/or microbial carriage in peripheral tissues; these mediators are also associated with translocation of PAMPs into circulation as a result of intestinal damage (plasma IFABP, stool AAT) and environmental enteropathy (9). We therefore investigated whether there was an association between inflammatory biomarker concentrations and antigen-specific immune function at 18 months of age. Children enrolled in the sub-study had similar concentrations of all systemic inflammatory and enteropathy biomarkers to the SHINE cohort (**Supplementary Table 2**) with the exception of plasma IFABP (median, interquartile range: 314.8 μg/mL (142.2; 588.5) versus 211.7 mg/mL (112.6; 400.3), p = 0.006).

We did not find evidence that plasma CRP or sCD14 at 18 months was associated with antigen-specific immune function (p > 0.05; **Supplementary Table 4**). However, biomarkers of enteropathy and intestinal damage were associated with higher concentration of antigen-specific immune mediators. The strongest association was with LPS-specific MPO, which increased by 0.44 pg/mL (95% CI: 1.06, 4.85, p = 0.035) in culture supernatants per 1-nmol/mL increase in stool neopterin concentration; this association was strengthened after adjustment for the SHINE arm, hub, child sex and maternal HIV status (adj. GMD (95% CI): 2.48 (1.19, 5.15), p = 0.015). There was also evidence for higher antigen-specific pro-inflammatory cytokine concentrations per unit increase in other biomarkers of environmental enteropathy (**Supplementary Table 4**): LPS-specific TNFα (GMD (95% CI), AAT: 0.41 (0.17, 0.98), p = 0.044; adj. GMD (95% CI): 0.36 (0.16, 0.81), p = 0.013), HKST-specific TNFα (GMD (95% CI), IFABP: 0.36 (0.17, 0.77), p = 0.009; adj. GMD (95%CI): 0.44 (0.23, 0.81), p = 0.009), HKST-specific IL-6 (GMD (95% CI), IFABP: 0.41 (0.18, 0.96), p = 0.040; not significant in adjusted models) and LPS-specific IL-8 (GMD (95%CI): 0.53 (0.27, 0.97), p = 0.062; adj. GMD (95% CI): 0.44 (0.24, 0.82), p = 0.009). These data suggest that concurrent enteropathy drives a heightened capacity to generate pro-inflammatory cytokine and pro-ROS responses to a new bacterial antigen stimulus *in vitro*.

### Relationship Between Maternal Health During Pregnancy and Infant Immune Function

Maternal health may contribute to development of infant immune function since maternal infections, inflammation and nutritional state during pregnancy influence the *in utero* environment, birthweight and breastmilk composition; to investigate this, we used baseline data collected from mothers during their pregnancies (**Table 3**). Mothers of children enrolled in the sub-study were taller (mean ± standard deviation: 161 ± 7 versus 160 ± 9 cm), and a slightly lower proportion were married (95.0% versus 95.2%) compared to the SHINE cohort; other maternal characteristics, including levels of systemic and intestinal inflammatory biomarkers, were similar (**Supplementary Table 2**). Eighteen mothers (16.2% versus 15% in SHINE) were HIV-positive, and of those with available data on treatment, all were taking ART and all but one were taking prophylactic cotrimoxazole during pregnancy. Twelve mothers (13.0%) had detectable *S. haematobium* ova in their urine.

**Table 3:** Baseline household and maternal characteristics of the SHINE immune function sub-study cohort.

Characteristics	Enrolled in the SHINE immune function sub-study
**Mothers, N*^a^***	111
**Maternal characteristics**	
Age, mean (SD) [n], years	27.0 (6.0) [91]
Height, mean (SD) [n], cm	161.1 (6.5) [108]
*Schistosoma haematobium* ova in stool, n/N (%)	12/92 (13.0%)
HIV status, n/N (%):	
Positive	18/111 (16.2%)
Negative	88/111 (79.3%)
Unknown	5/111 (4.5%)
CD4 count, mean (SD) [n], cells/mL	625.6 (249.1) [16]
Antiretroviral therapy (ART) during pregnancy, n/N (%)	16/16 (100.0%)
Prophylactic cotrimoxazole during pregnancy, n/N (%)	12/13 (92.3%)
Systemic inflammation:	
Plasma C-reactive protein (CRP), median (IQR) [n], ng/mL	2,748 (1,074; 7,169) [62]
Plasma soluble (s)CD14, median (IQR) [n], pg/mL	1,065,818 (837,107; 1,337,835) [75]
Intestinal inflammation:	
Stool myeloperoxidase (MPO), median (IQR) [n], ng/mL	800 (800; 1,088) [83]
Stool neopterin, median (IQR) [n], nmol/L	41.2 (35; 71) [83]

^a^The cohort includes one set of twins.

There was limited evidence for an association between maternal age, anthropometry (height, MUAC), HIV status or *S. haematobium* infection status at baseline and the antigen-specific immune function of their children at 18 months of age (p > 0.05; data not shown). However, maternal plasma sCD14 concentrations were associated with higher LPS- and HKST-specific TNFα (GMD (95% CI), LPS: 20.90 (1.75, 247.00), p = 0.016; HKST: 7.92 (1.99, 31.80), p = 0.003), LPS-specific IL-6 (GMD (95% CI): 7.69 (1.36, 43.80), p = 0.021) and HKST-specific IL-8 (GMD (95% CI): 4.44 (1.06, 18.72), p = 0.041). Adjustment for the SHINE arm, maternal age, HIV status and *S. haematobium* infection status weakened the association between maternal sCD14 and 18-month antigen-specific immune mediators; evidence for an association with 18-month HKST-specific TNFα was retained in adjusted models (adj. GMD (95% CI): 7.10 (1.51, 33.10), p = 0.013). Higher plasma CRP (GMD (95% CI): 1.80 (1.08, 2.97), p = 0.023; adj. GMD (95% CI): 1.73 (1.03, 2.92), p = 0.037) and stool neopterin (GMD (95% CI): 2.48 (1.40, 4.44), p = 0.002; adj. GMD (95% CI): 2.32 (1.32, 4.10), p = 0.003) concentrations during pregnancy were also associated with higher HKST-specific IL-8 responses in children at 18 months in both adjusted and unadjusted models. Thus, maternal inflammation and enteropathy during pregnancy influence the antibacterial immune function of their children at 18 months.

### Impact of Household WASH and IYCF Interventions on Antibacterial Immune Function

The rationale behind the SHINE trial was that improved household WASH and IYCF during early life would improve child linear growth and reduce stunting; we hypothesised that the SHINE interventions might also influence antibacterial immune cell function. During pregnancy and prior to initiation of the SHINE interventions, WASH access and behaviours were limited and only a minority of households met the minimum dietary diversity scores (**Supplementary Table 2**), as is typical of rural households in this region. Compared to the larger SHINE cohort, households of children enrolled in the sub-study were similar across WASH indices with the exception of fewer households having access to handwashing stations with rubbing agent (0% versus 0.7%; **Supplementary Table 2**). Similar proportions of households in the sub-study and SHINE cohorts met minimum dietary diversity scores at baseline (35.4% versus 39.7%; **Supplementary Table 2**).

Consistent with SHINE primary outcome measures (19, 21), we did not identify a significant interaction between the effects of the SHINE WASH and IYCF interventions on unstimulated or antigen-specific outcome variables (**Supplementary Table 5**); we therefore compared WASH (i.e., children exposed to the SHINE WASH or WASH+IYCF arm) to no WASH (i.e., children exposed to the SHINE IYCF or SOC arm; **Supplementary Table 6**) and IYCF (i.e., children exposed to the SHINE IYCF or WASH+IYCF arm) to no IYCF (i.e., children exposed to the SHINE WASH or SOC arm; **Supplementary Table 7**). The WASH arm had a higher proportion of stunted children at 18 months than the no WASH arm (51.5% versus 33.8%; p = 0.069; **Supplementary Table 6**). We therefore included stunting status with the SHINE hub in adjusted tobit regression of immune function by the SHINE arm.

**Figure 4:**
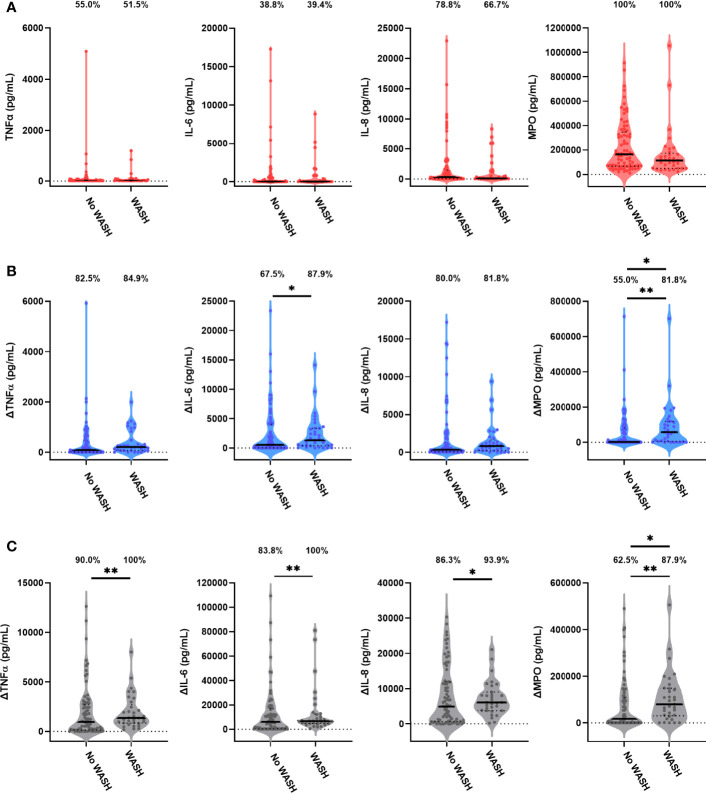
Unstimulated and bacterial antigen-stimulated immune-mediator production by blood immune cells from children exposed versus not exposed to a household WASH intervention. Violin plots (median and interquartile range indicated) of **(A)** unstimulated (red), **(B)** LPS-stimulated (blue) and **(C)** HKST-stimulated (grey) TNFα, IL-6, IL-8 and MPO concentrations in 24h culture supernatants. LPS- and HKST-specific concentrations (Δ) were calculated for each child by subtracting concentrations present in matched unstimulated culture supernatants. Proportions indicate participants with mediator concentration > ELISA limit of detection **(A)** and participants with antigen-stimulated mediator concentration > unstimulated **(B, C)**. Proportions of children with detectable mediator levels and mediator concentrations were compared by exposure to the SHINE WASH intervention (WASH n = 33, no WASH n = 80) *via* multinomial logit regression and censored log-normal (tobit) regression, respectively (unadjusted analyses indicated; full results in **Table 4**); *p < 0.05, **p < 0.01.

Children in the WASH arm had lower unstimulated MPO concentrations than those in the no WASH arm (**Figure 4A**; **Table 4**). Neither proportions nor concentrations of other mediators in unstimulated cultures differed by the WASH arm (**Figure 4A**; **Table 4** **;** **Supplementary Table 8**). Higher proportions of children in the WASH versus no WASH arm had detectable bacterial antigen-specific responses; this effect was strongest for LPS-specific MPO (logit regression OR (95% CI): 3.67 (1.28, 10.59), p = 0.016) and HKST-specific MPO (OR (95% CI): 4.35 (1.42, 13.33), p = 0.010; **Figures 4B, C**). LPS- and HKST-specific IL-6 and MPO and HKST-specific TNFα and IL-8 concentrations were also higher in the WASH versus no WASH arm in unadjusted models (**Figures 4B, C**). This pattern was retained for LPS- and HKST-specific MPO after adjustment for the SHINE hub and stunting status (**Table 4**), suggesting that the WASH intervention had a greater total effect on antibacterial MPO production than on pro-inflammatory cytokines.

**Table 4:** Censored log-normal (tobit) regression analysis of the relationship between WASH intervention arm and immune-mediator production at 18 months of age.

Outcome	Unadjusted			Adjusted for SHINE hub and 18-month stunting status		
	GMD*^a^*	95% CI	p	Adj. GMD*^a^*	Adj. 95% CI	p
**Unstimulated * ^b^ *:**						
TNFα	1.11	0.46; 2.64	0.824	1.20	0.70; 2.01	0.514
IL-6	1.28	0.07; 25.02	0.868	1.25	0.11; 13.87	0.860
IL-8	0.45	0.16; 1.40	0.176	0.63	0.26; 1.49	0.289
MPO	0.73	0.48; 1.12	0.142	**0.58**	**0.40; 0.86**	**0.007**
**Antigen-specific * ^c^ *:**						
LPS-specific TNFα	3.74	0.52; 26.82	0.189	1.88	0.74; 4.76	0.182
HKST-specific TNFα	**4.81**	**1.54; 15.03**	**0.007**	1.80	0.97; 3.39	0.062
LPS-specific IL-6	**4.48**	**1.04; 19.10**	**0.04**	1.67	0.55; 5.05	0.368
HKST-specific IL-6	**4.53**	**1.49; 13.87**	**0.008**	1.65	0.86; 3.16	0.133
LPS-specific IL-8	1.55	0.44; 5.47	0.494	0.61	0.36; 2.51	0.920
HKST-specific IL-8	**4.39**	**1.23; 15.63**	**0.023**	1.65	0.70; 3.90	0.257
LPS-specific MPO	**10.48**	**1.84; 60.31**	**0.008**	**3.74**	**1.20; 11.59**	**0.023**
HKST-specific MPO	**5.10**	**1.77;14.88**	**0.003**	**4.67**	**1.58;13.87**	**0.005**

^a^Geometric mean difference in mediator concentration (pg/mL) between the WASH group (n = 33) and the no WASH group (n = 80) estimated from censored log-normal (tobit) regression coefficient; bolded text indicates mediators with evidence for an association with the WASH intervention (p < 0.05).

^b^Concentrations in unstimulated whole blood culture supernatants.

^c^ΔConcentrations between antigen-stimulated and unstimulated whole blood culture supernatants.

**Figure 5:**
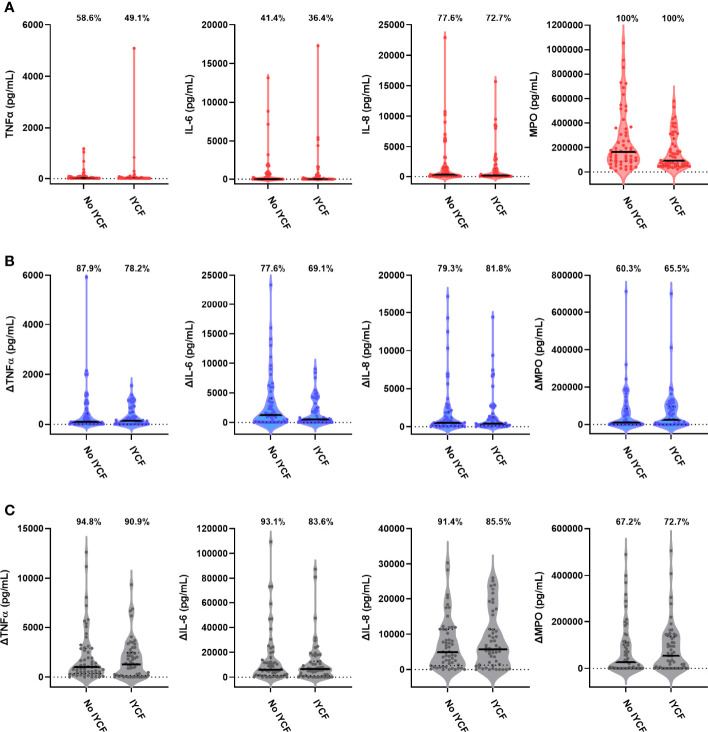
Unstimulated and bacterial antigen-stimulated immune-mediator production by blood immune cells from children exposed versus not exposed to a household IYCF intervention. Violin plots (median and interquartile range indicated) of **(A)** unstimulated (red), **(B)** LPS-stimulated (blue) and **(C)** HKST-stimulated (grey) TNFα, IL-6, IL-8 and MPO concentrations in 24h whole blood culture supernatants. LPS- and HKST-specific concentrations (Δ) were calculated for each child by subtracting concentrations present in matched unstimulated culture supernatants. Proportions indicate participants with mediator concentration > ELISA limit of detection **(A)** and participants with antigen-stimulated mediator concentration > unstimulated **(B, C)**. Proportions of children with detectable mediator levels and mediator concentrations were compared between children exposed to the SHINE household IYCF intervention (n = 55) and those not exposed to the IYCF intervention (n = 58) by multinomial logit regression and censored log-normal (tobit) regression, respectively (unadjusted analyses indicated; full results in **Table 5**).

We found no evidence for an effect of the SHINE IYCF intervention on proportions or concentrations of unstimulated or bacterial antigen-specific mediators (**Figure 5**; **Table 5** **;** **Supplementary Table 8**).

**Table 5:** Censored log-normal (tobit) regression analysis of the relationship between IYCF intervention arm and immune-mediator production at 18 months of age.

Outcome	Unadjusted			Adjusted for SHINE hub and 18-month stunting status		
	GMD*^a^*	95% CI	p	Adj. GMD*^a^*	Adj. 95% CI	p
**Unstimulated * ^b^ *:**						
TNFα	0.65	0.29; 1.46	0.300	0.79	0.50; 1.27	0.341
IL-6	0.59	0.04; 9.49	0.710	0.70	0.08; 6.49	0.758
IL-8	0.81	0.29; 2.27	0.690	0.87	0.39; 1.93	0.724
MPO	0.73	0.49; 1.07	0.104	0.75	0.53; 1.06	0.102
**Antigen-specific * ^c^ *:**						
LPS-specific TNFα	0.25	0.04; 1.68	0.156	0.68	0.28; 1.65	0.392
HKST-specific TNFα	1.92	0.17; 1.62	0.259	0.76	0.43; 1.34	0.332
LPS-specific IL-6	0.32	0.08; 1.30	0.110	0.52	0.20; 1.22	0.188
HKST-specific IL-6	0.55	0.18; 1.65	0.284	0.79	0.44; 1.42	0.436
LPS-specific IL-8	0.78	0.24; 2.53	0.674	0.87	0.36; 2.07	0.754
HKST-specific IL-8	0.83	0.23; 2.92	0.769	1.09	0.50; 2.43	0.819
LPS-specific MPO	1.65	0.31; 8.58	0.556	3.97	0.52; 4.10	0.468
HKST-specific MPO	1.73	0.62; 4.85	0.295	5.00	0.67; 5.05	0.235

^a^Geometric mean difference in mediator concentration (pg/mL) between the IYCF group (n = 55) and the no IYCF group (n = 58) estimated from censored log-normal (tobit) regression coefficient.

^b^Concentrations in unstimulated whole blood culture supernatants.

^c^ΔConcentrations between antigen-stimulated and unstimulated whole blood culture supernatants.

## Discussion

In this study, we directly assessed the capacity for blood immune cells from 113 children in rural Zimbabwe to respond to bacterial antigen challenge *in vitro*—a model for how each child might respond to a newly acquired bacterial infection. Antibacterial immune function was highly heterogeneous even among this predominantly healthy HIV-negative cohort. We found evidence that stunting status and environmental enteropathy at 18 months of age, maternal inflammation during pregnancy, birthweight and exposure to improved household WASH contributed to inter-individual variation that could plausibly contribute to differences in infectious susceptibility.

Stunting is a multifactorial syndrome arising among children exposed to a marginal diet, recurrent infections, chronic inflammation and impaired gut function. In support of our hypothesis that stunting alters antibacterial immune function, higher proportions of stunted children responded to bacterial antigen challenge and these children produced higher concentrations of LPS-specific pro-inflammatory cytokines (IL-6 and IL-8) than children who were non-stunted. Epigenetic analyses of 8 stunted and 11 non-stunted Bangladeshi children have previously identified a histone-3-lysine-4 methylation signature on blood immune cells that could putatively affect their function (29). However, this is the first study to our knowledge to directly characterise the relationship between antibacterial immune cell function and stunting (6, 7). Stunting and wasting are indicators of undernutrition which frequently coexist; wasting tends to be more acute and reversible than stunting (30). Several small studies have previously assessed immune cell function in wasted children, among whom cytokine responses to *in vitro* activation tended to be lower compared to well-nourished controls (31, 32) or to their own responses after nutritional recovery (32, 33). More recently, a study among severely unwell 2–23-month-old children in Kenya and Uganda with a range of wasting severities found evidence that hospitalised children had consistently higher LPS-specific pro-inflammatory mediator concentrations within 48 h of admission (n = 43), on the day of discharge (n = 60) and, for survivors, 6 months after admission (n = 51) than healthy community controls (n = 41) (34). In contrast to LPS (a TLR4 ligand), mediator responses to TLR7/8 ligands and Staphylococcal enterotoxin B, which activates innate and adaptive immune cells by binding MHC-II and T-cell receptor molecules, were lower in unwell children versus controls (34). Our observations among stunted children are consistent with those for TLR4-driven mediator responses among wasted children, implicating priming of the pro-inflammatory innate immune response to gram-negative bacteria among children with both acute and chronic forms of undernutrition. Our study recruited children from the community which minimised immune heterogeneity due to severe symptomatic infections, associated clinical complications and antibiotic treatment which are common among hospital inpatients. It is plausible that the differences we observed in antigen-specific mediator responses are due to differences in circulating immune cell composition between stunted and non-stunted children; to our knowledge, there are no published studies comparing blood immunophenotypes between stunted and non-stunted children from the same community. We did not localise the cellular source of supernatant mediators; however, our choice of short culture time, PAMPs and ELISA favours assessment of innate cell types, particularly monocytes. The expression of TLR2 and TLR4, the primary receptors for LPS and HKST, could also be influential. For example, low LPS-specific TNFα and IL-6 production was related to low monocyte TLR4 expression among 20 uninfected infants born preterm in Austria (35). A more granular evaluation of cellular antibacterial immune responses in stunting is warranted.

Immune function was not associated with LAZ at 3, 6, 12 or 18 months or with the IYCF intervention, which improved linear growth and reduced stunting prevalence in the SHINE trial (19, 21). In contrast to severely unwell children in Kenya and Uganda, among whom MUAC was positively associated with LPS-specific responses at hospital discharge (34), we did not identify an association between antigen-specific immune function and MUAC or with other indicators of concurrent wasting (WHZ, WAZ); this may reflect the fact that our cohort was largely within healthy WHZ and WAZ ranges. Thus, our data implicate factors besides anthropometry and feeding-based interventions (such as IYCF) in the differences in immune function between stunted and non-stunted children. Enteropathogen carriage is an *in vivo* source of PAMPs, greater among children with slower growth velocities and associated with systemic and intestinal inflammation (13, 16, 36). Circulating LPS (also termed endotoxin) is the most studied PAMP in undernourished cohorts and widely used as an indicator of increased translocation of commensal bacteria and enteropathogens from the gut (9). The LPS-TLR4 pathway is also a means by which circulating monocyte function is modified in gram-negative bacterial sepsis (37), which may contribute to the infectious susceptibility that characterises post-sepsis convalescence. Consistent with pathogen carriage contributing to higher antibacterial mediator production, the effect of stunting status on LPS-specific pro-inflammatory cytokines was reduced when the small number children with recently reported symptoms of infection were excluded in sensitivity analyses. Asymptomatic pathogen carriage and LPS concentrations were not assessed in this study but could also plausibly underlie a more primed circulating immune cell response to PAMP. We found some evidence for this in the association between 18-month biomarkers of environmental enteropathy (stool neopterin, MPO and AAT, plasma IFABP), particularly between LPS-specific MPO and stool neopterin. Stool MPO and plasma IFABP were also associated with seroconversion in response to the oral rotavirus vaccine in the SHINE rotavirus sub-study (38). Consistent with humoral responses to the rotavirus vaccine (38), we did not find evidence for an association between antigen-specific immune-mediator concentrations and plasma sCD14, a biomarker of monocyte activation frequently used as an indirect indicator of microbial translocation. Thus, antibacterial functions of circulating immune cells appear to be more influenced by intestinal than systemic inflammation at 18 months; this could reflect age-related changes in the gut to limit systemic inflammation in the context of microbial colonisation and recurrent infections at earlier ages. In the SHINE trial, enteropathy biomarkers changed significantly between the 1- and 18-month visits (23); stool MPO and neopterin tended to be lower at 18 months, indicative of reduced innate immune cell accumulation in the lamina propria in older children. In a Zambian cohort of children under 5 years of age, stunting was associated with cumulative adaptation of the gastrointestinal tract to reduce LPS translocation with age (9).

Since both stunting and immune development have *in utero* origins, a strength of this study was the availability of longitudinal data on each child’s early life with which to explore factors that could contribute to the heterogeneity in immune function we observed at 18 months. Direct indicators of baseline maternal health were not associated with 18-month immune function. However, antigen-specific MPO was higher among children with higher birthweight, indicating that children born smaller may have a lower capacity to produce this enzyme even when born within the healthy birthweight range. Furthermore, children born to mothers with higher concentrations of systemic (CRP and sCD14) and intestinal (neopterin) inflammatory biomarkers during pregnancy had higher pro-inflammatory cytokine responses to bacterial antigens at the 18-month visit. The most consistent positive relationship was with maternal sCD14, an LPS receptor/TLR4 co-receptor expressed by circulating monocytes that is shed upon their activation (39). We did not find evidence for an association between 18-month concentrations of this biomarker and immune function in children, suggesting that history of exposure to sCD14 *in utero* has more influence on a child’s capacity to produce pro-inflammatory cytokines in response to bacteria than their concurrent circulating sCD14 concentrations. *In utero* exposure to maternal monocyte activation could prime responses to gram-negative bacteria *via* a variety of mechanisms. For example, since monocytes are early responders to circulating PAMPs *in vivo (* 39), sCD14 could be an indicator of microbial translocation during pregnancy with implications for *in utero* growth; among the 207 SHINE mothers for whom stool samples were analysed by metagenomic sequencing, microbiome taxa and metabolic genes were predictive of birthweight (40). We adjusted analyses by maternal HIV and *S. haematobium* but did not screen for other potentially influential pathogens during pregnancy. Maternal sCD14 could also reduce infant LPS exposure prior to weaning; radiolabelled sCD14 from rat dams persists in the small intestine and blood of their pups up to 8 h after breastmilk ingestion (41).

In light of the relationships identified between antibacterial immune function and child stunting, enteropathy and maternal inflammation during pregnancy, all plausibly mediated through enteropathogen exposure, we went on to characterise the effects of household WASH. WASH is posited as an intervention to reduce child stunting *via* reducing enteropathogen exposure (42); however, the SHINE WASH intervention did not improve linear growth at 18 months in the main trial (19, 21) nor did it effect diarrhoea or enteropathy biomarkers (21, 23, 38), suggesting that more transformative WASH (e.g., piped water, improved housing, managed sanitation) is required to sufficiently reduce enteropathogen exposure for meaningful impacts on child growth outcomes (43). However, since immune cells are direct responders to PAMPs, we hypothesised that they would be more sensitive to small changes in enteropathogen exposure than growth outcomes or indirect biomarker measures. Accordingly, exposure to the WASH intervention was associated with higher proportions and concentrations of LPS- and HKST-specific MPO. MPO is more abundantly expressed in neutrophils than other blood immune cells and catalyses production of ROS, a potent microbicidal response to infection (27). Whilst we did not directly measure the ability of blood leukocytes to kill bacteria, these data suggest that exposure to improved household WASH in early life may increase neutrophil-mediated antibacterial defence. The observation that children exposed to the WASH intervention had increased cellular immunoreactivity to bacterial PAMPs is consistent with findings for humoral immune function in the SHINE rotavirus sub-study where there was a significant increase in seroconversion to the oral rotavirus vaccine among children in the WASH (n = 109) versus no WASH arm (n = 219) (44).

Our results should be interpreted in the context of several study limitations. Most notably, we did not have preexisting data on immune function in stunted versus non-stunted children with which to estimate sample size; given the heterogeneity in mediator concentrations, a larger sample size would have given greater power to detect differences in causal inference models. Cross-sectional assessment meant that longevity and clinical relevance of the pg/mL magnitude differences observed in immune function variables are unclear and longitudinal assessment is warranted (1, 6); production of higher concentrations of pro-inflammatory cytokines could benefit stunted children by promoting more effective clearance of bacterial infection but could also exacerbate immunopathology. For example, biomarker studies among severely wasted children requiring hospital admission indicate that pro-inflammatory cytokine concentrations are higher in plasma from children who die versus survive (45, 46) and nutritional recovery is slower among children with systemic inflammation (47). Inclusion of SHINE participants in this sub-study was based on blood sample volume and completion of an 18-month follow-up visit, meaning that our cohort reflects a healthier group of children than that reflected by the SHINE cohort as a whole and differences were evident between the intervention groups. Thus, further studies of the interrelationships between immune function, child growth and more direct measures of enteropathogen exposure and microbial translocation are required, particularly among children most vulnerable to adverse infectious outcomes.

In conclusion, this study provides proof of principle that stunted children have distinct antibacterial immune function to non-stunted children which is shaped by indicators of maternal and child enteropathogen exposure and inflammation to a greater extent than their anthropometry alone. A pertinent outstanding question is whether the differences that we identify in immune function are adaptive, benefitting health and survival in the context of adverse exposures, or deleterious, increasing risk of acquiring and succumbing to infection; answering this question will rely on combining longitudinal immune function assessment with clinical outcome measures among undernourished children (1, 6). Our results also demonstrate how assays of immune cell function could provide a sensitive tool with which to evaluate interventions targeting infectious morbidity and mortality among children growing up in LMIC.

## Data Availability Statement

The raw data supporting the conclusions of this article will be made available by the authors, without undue reservation.

## Ethics Statement

The studies involving human participants were reviewed and approved by the Medical Research Council of Zimbabwe Institutional Review Board of the Johns Hopkins Bloomberg School of Public Health. Written informed consent to participate in this study was provided by the participants’ legal guardian/next of kin.

## Author Contributions

KM and JT contributed equally to this work. KM assisted CB to develop the laboratory assays and managed laboratory operations for the sub-study with support from SR. JT undertook statistical analyses supervised by CB and with support from RN and BC. FM and CC performed whole blood cultures in field laboratories. SR, MG, and PM performed ELISA. NT managed field operations, KM managed the laboratory, FM supervised data collection nurses, BM supervised the field data supervisors, and RN developed and managed information technology for the main SHINE trial. JH conceived of SHINE and secured funding for the main trial. AP directed clinical and laboratory aspects of SHINE and secured funding for the sub-study. CB designed and managed the sub-study and prepared the first draft of the manuscript. All authors contributed to the article and approved the submitted version.

## Funding

The SHINE trial was funded by the Bill and Melinda Gates Foundation (OPP1021542 and OPP1143707), the United Kingdom Department for International Development (DFID/UKAID), Wellcome Trust (093768/Z/10/Z and 108065/Z/15/Z), Swiss Agency for Development and Cooperation, and UNICEF (PCA-2017-0002). CB is funded by a Sir Henry Dale Postdoctoral Research Fellowship from the Wellcome Trust and The Royal Society (206225/Z/17/Z). Open Access Publication was funded *via* Wellcome.

## Author Disclaimer

The study funders were not involved in data collection, analysis, interpretation, or manuscript preparation.

## Conflict of Interest

The authors declare that the research was conducted in the absence of any commercial or financial relationships that could be construed as a potential conflict of interest.

## Publisher’s Note

All claims expressed in this article are solely those of the authors and do not necessarily represent those of their affiliated organizations, or those of the publisher, the editors and the reviewers. Any product that may be evaluated in this article, or claim that may be made by its manufacturer, is not guaranteed or endorsed by the publisher.
