# Supplementary material for: Stunting Status and Exposure to Infection and Inflammation in Early Life Shape Antibacterial Immune Cell Function Among Zimbabwean Children
Source: Front Immunol. 2022 Jun 13;13:899296. doi: 10.3389/fimmu.2022.899296 (PMC9234645; doi:10.3389/fimmu.2022.899296)
Supplement: Supplementary file 1 [file DataSheet_1.docx]

**Stunting status and exposure to infection and inflammation in early life shape anti-bacterial immune cell function among Zimbabwean children**

**Authors:** Kuda Mutasa^1†^; Joice Tome^1†^, Sandra Rukobo^1^; Margaret Govha^1^; Patience Mushayanembwa^1^; Farai S. Matimba^1^; Courage K. Chiorera^1^; Florence D. Majo^1^; Naume V. Tavengwa^1^; Batsirai Mutasa^1^; Bernard Chasekwa^1^; Jean H. Humphrey^1,2^; Robert Ntozini^1^; Andrew J. Prendergast^1,3^; Claire D. Bourke^1,3^* on behalf of the SHINE Trial Team

^†^These authors have contributed equally to this work and share first authorship

**Affiliations:**

^1^Zvitambo Institute for Maternal and Child Health Research, Harare, Zimbabwe

^2^Department of International Health, Johns Hopkins Bloomberg School of Public Health, Baltimore MD, USA

^3^Centre for Genomics and Child Heath, Queen Mary University of London, London, UK

***Corresponding author:** Claire D. Bourke; [c.bourke@qmul.ac.uk](mailto:c.bourke@qmul.ac.uk); Centre for Genomics and Child Heath, Blizard Institute, Newark Street, Queen Mary University of London, E1 2AT, London, UK

**Running title:** Immune function of Zimbabwean children

**Keywords:** Immune function; Inflammation; Stunting; Child Health; Pregnancy; Water Sanitation and Hygiene (WASH); Enteropathogens; Cytokines; Bacteria; Endotoxin; Zimbabwe

**SUPPLEMENTARY INFORMATION**

**SUPPLEMENTARY FIGURES:**

**Supplementary Figure 1:** Comparison of immune mediator concentrations in unstimulated whole blood culture supernatants by 18-month stunting status and SHINE intervention arm.

**Supplementary Figure 2:** LPS-specific immune mediator production grouped by child HIV-exposure, anaemia and symptom status at 18 months.

**Supplementary Figure 3:** HKST-specific immune mediator production grouped by child HIV-exposure, anaemia and symptom status at 18 months.

**Supplementary Figure 4:** Relationship between birthweight and bacterial antigen-stimulated immune mediator production at 18 months.

**SUPPLEMENTARY TABLES:**

**Supplementary Table 1:** Summary of causal inference models for the effects of early life exposure variables on 18-month anti-bacterial immune cell function outcome variables

**Supplementary Table 2:** Comparison of household, maternal & infant characteristics of the immune function sub-study cohort versus SHINE participants not enrolled in the sub-study

**Supplementary Table 3:** Censored log-normal (tobit) regression analysis of the relationship between stunting status and unstimulated concentrations of IL-12p70, Hepcidin, sCD163 and IFNβ at 18 months of age

**Supplementary Table 4:** Censored log-normal (tobit) regression analysis of the relationship between biomarkers of systemic inflammation, enteropathy and anti-bacterial immune function at 18 months of age

**Supplementary Table 5:** Censored log-normal (tobit) regression analysis of the effect of the interaction between WASH and IYCF intervention arms immune mediator production at 18 months of age

**Supplementary Table 6:** Characteristics with evidence for a difference between WASH and no WASH arms in the immune function sub-study cohort

**Supplementary Table 7:** Characteristics with evidence for a difference between IYCF and no IYCF arms in the immune function sub-study cohort

**Supplementary Table 8:** Censored log-normal (tobit) regression analysis of the relationship between the WASH and IYCF intervention arms and concentrations of IL-12p70, Hepcidin, sCD163 and IFNβ at 18 months of age

**SUPPLEMENTARY REFERENCES**

**
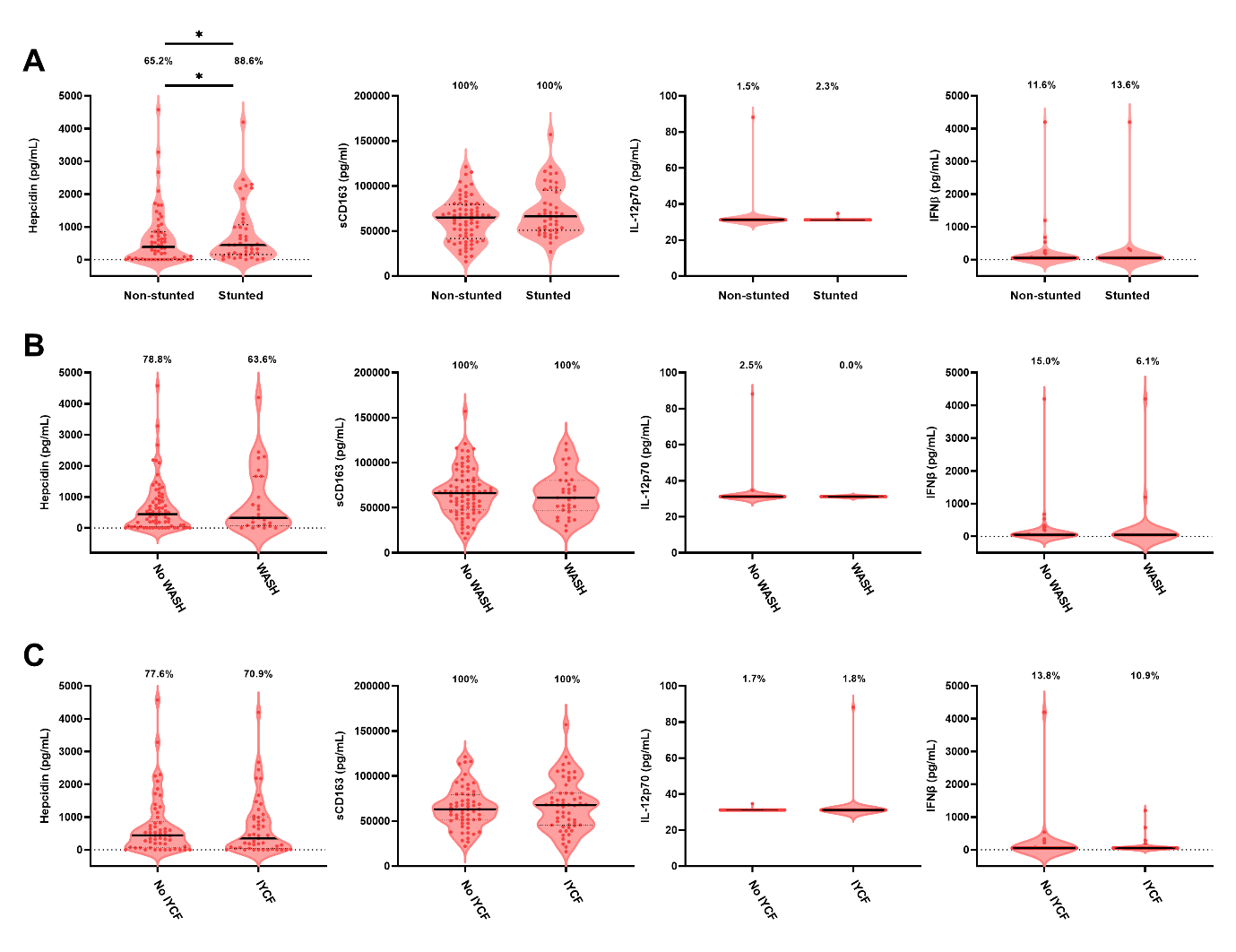
**

**Supplementary Figure 1. Comparison of immune mediator concentrations in unstimulated whole blood culture supernatants by 18-month stunting status and SHINE intervention arm.** Violin plots (median and interquartile range indicated) of hepcidin, soluble (s)CD163, IL-12p70 and IFNβ concentrations present in unstimulated culture supernatants. Proportions indicate participants with mediator concentration>ELISA limit of detection. Proportions of children with detectable mediator levels and mediator concentrations were compared by (A) 18-month stunting status (stunted n=44 stunted; non-stunted n=69), (B) exposure to the SHINE household WASH intervention (WASH n=33, no WASH n=80) and (C) exposure to the SHINE household IYCF intervention (IYCF n=55; no IYCF n=58) via multinomial logit regression and censored log-normal (tobit) regression, respectively (unadjusted analyses indicated); *p<0.05. Unstimulated TNFα, IL-6, IL-8 and MPO data are plotted by stunting status in **Figures 3**, WASH intervention in **Figure 4** and IYCF intervention in **Figure 5**.


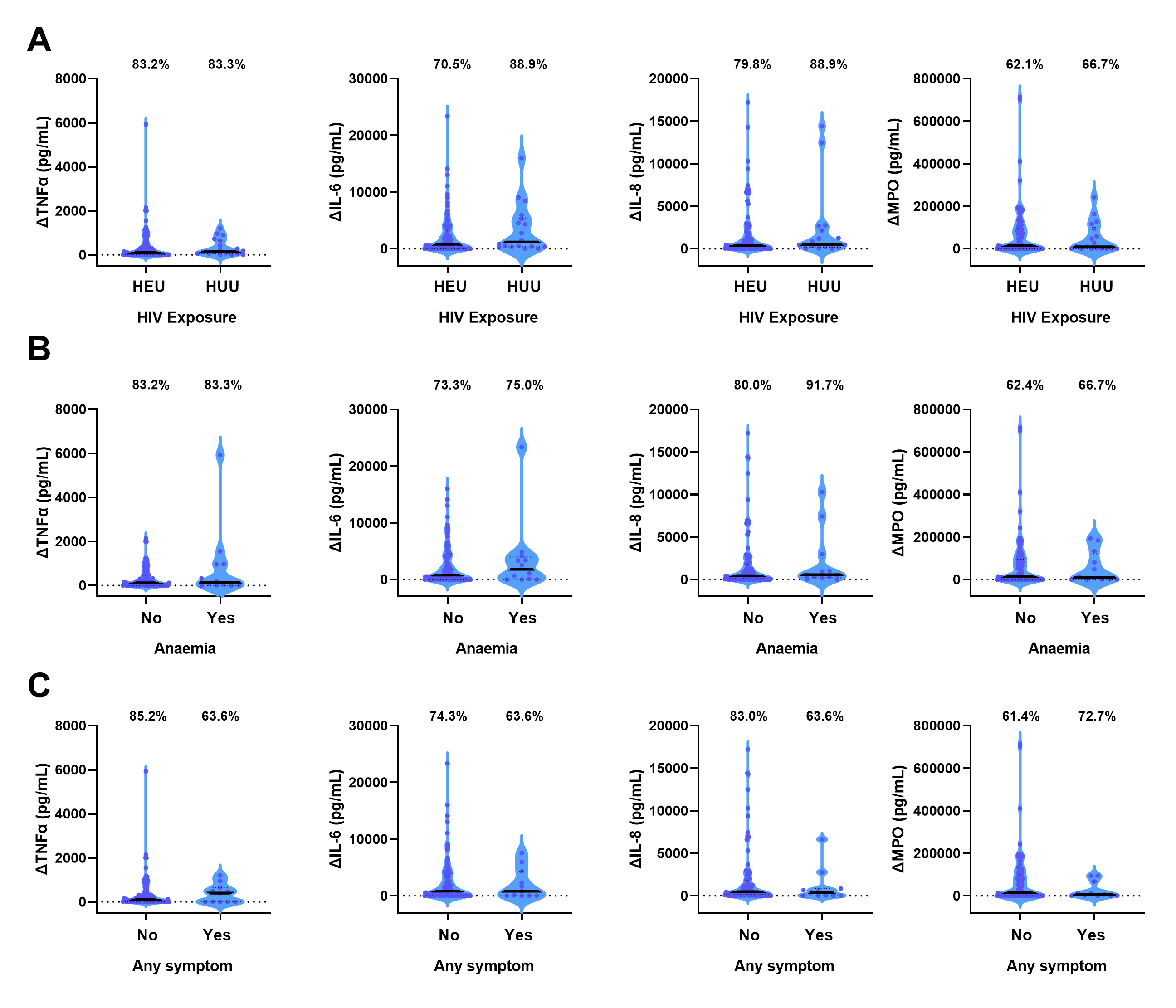


**Supplementary Figure 2. LPS-specific immune mediator production grouped by child HIV-exposure, anaemia and symptom status at 18 month****s.** Violin plots of antigen-specific TNFα, IL-6, IL-8 and MPO concentrations present in supernatants stimulated with LPS after subtraction of concentrations present in matched unstimulated culture supernatants (Δ). Mediators are grouped by children’s (A) exposure to maternal HIV (HUU – HIV unexposed uninfected, n=95; HEU - HIV exposed uninfected, n=18); (B) 18-month anaemia status (haemoglobin<10.5g/dL; Yes, n=12; No, n=111); and, (C) any caregiver-reported symptom of infection reported in the 7 days prior to the 18-month visit (Yes, n=11; No, n=101). Proportions indicate participants with antigen-stimulated mediator concentration> unstimulated. Due to low participant numbers in case groups (<10 events per group), the modifying effects of these variables on the relationship between immune function and stunting status at 18 months were evaluated in sensitivity analyses rather than by inclusion in adjusted tobit regression models (**Table 2**).


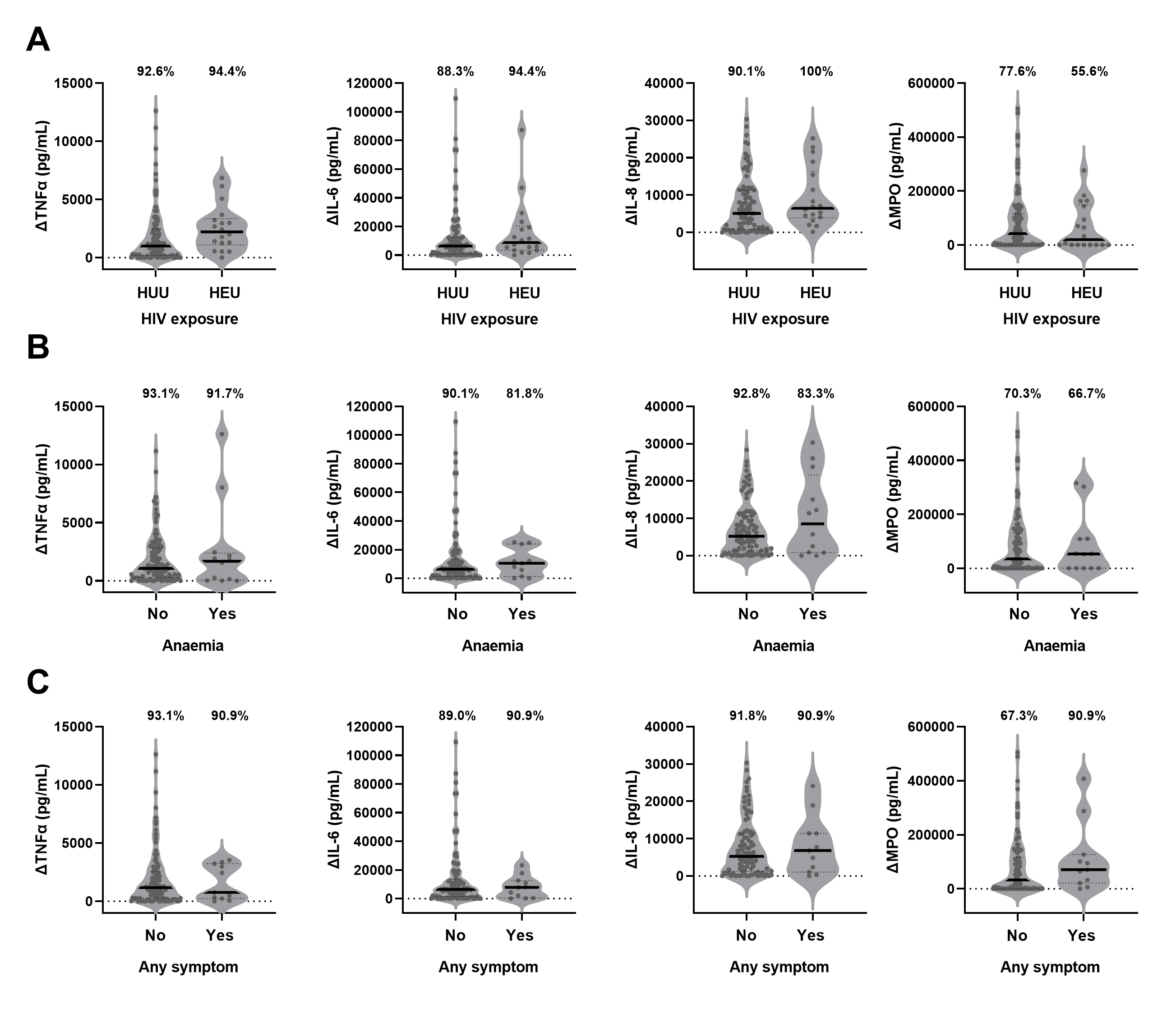


**Supplementary Figure 3. HKST-specific immune mediator production grouped by child HIV-exposure, anaemia and symptom status at 18 months.** Violin plots of antigen-specific TNFα, IL-6, IL-8 and MPO concentrations present in supernatants stimulated with HKST after subtraction of concentrations present in matched unstimulated culture supernatants (Δ). Mediators are grouped by children’s (A) exposure to maternal HIV (HUU – HIV unexposed uninfected, n=95; HEU - HIV exposed uninfected, n=18); (B) 18-month anaemia status (haemoglobin<10.5g/dL; Yes, n=12; No, n=111); and, (C) any caregiver-reported symptom of infection reported in the 7 days prior to the 18-month visit (Yes, n=11; No, n=101). Proportions indicate participants with antigen-stimulated mediator concentration>unstimulated. Due to low participant numbers in case groups (<10 events per group), the modifying effects of these variables on the relationship between immune function and stunting status at 18 months were evaluated in sensitivity analyses rather than by inclusion in adjusted censored log-normal (tobit) regression models (**Table 2**).


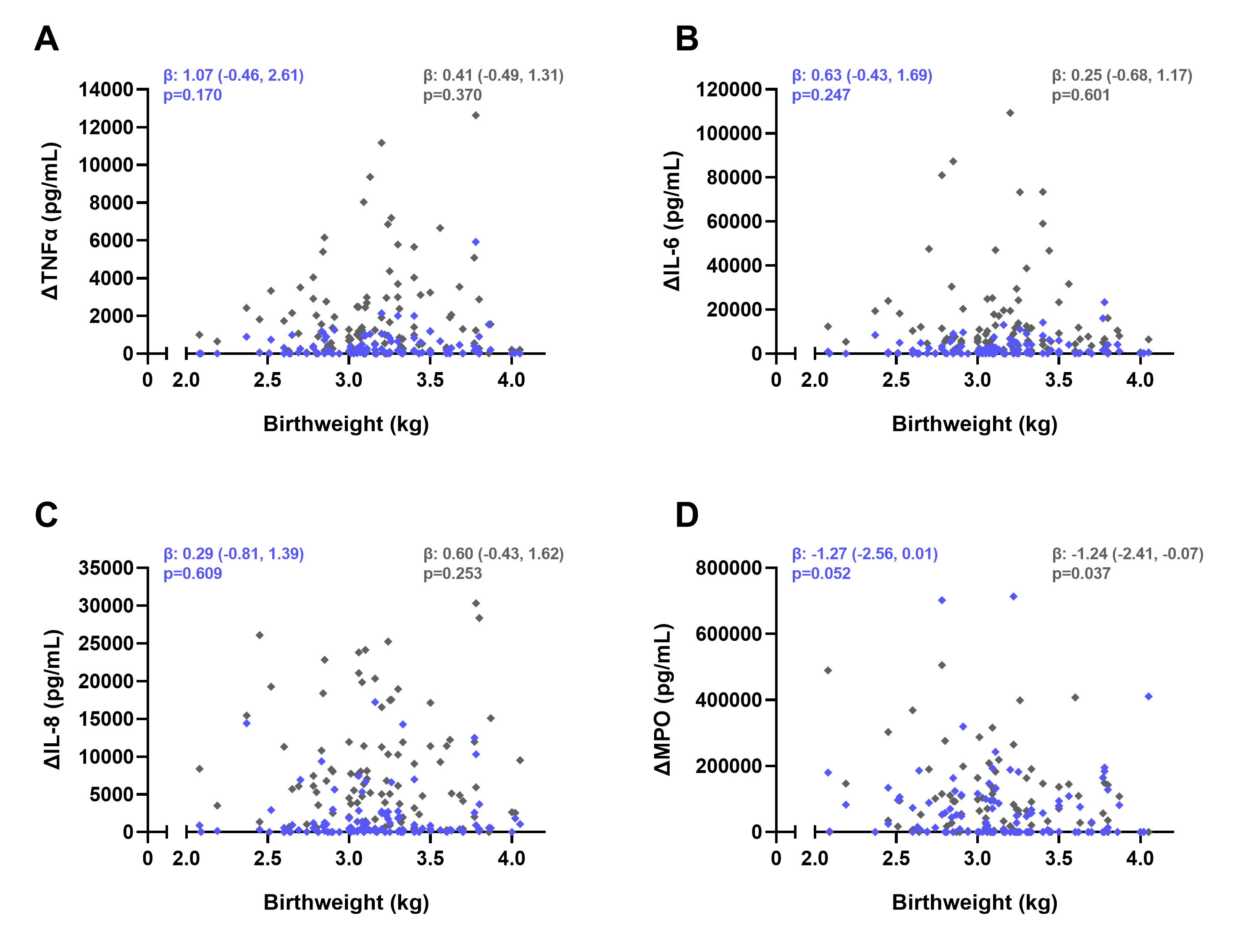


**Supplementary Figure 4: Relationship between birthweight and bacterial antigen-stimulated immune mediator production at 18 months.** Scatter plots of (A) TNFα; (B) IL-6; (C) IL-8; and (D) MPO concentration present in supernatants stimulated with LPS (blue) or HKST (grey) after subtraction of concentration present in matched unstimulated culture supernatants (Δ) plotted against birthweight. Results of unadjusted censored log-normal (tobit) regression are shown (n=111).

**Supplementary Table 1: Summary of causal inference models for the effects of early life exposure variables on 18-month anti-bacterial immune cell function outcome variables**

| **Model** | **Exposure^1^** | **Potential confounders** | **Approach** |
| --- | --- | --- | --- |
| **18-month stunting** | 18-month stunting status | Infant sex^2^ | Factor in adjusted models |
|  |  | Birthweight^3^ | Factor in adjusted models |
|  |  | HIV-exposure^2^ | Sensitivity analysis |
|  |  | Anaemia status^2^ | Sensitivity analysis |
|  |  | Symptoms of infection^2^ | Sensitivity analysis |
|  |  | SHINE hub^4^ | Factor in adjusted models |
|  |  | SHINE intervention^4^ | Factor in adjusted models |
|  |  | SHINE cluster^5^ | Included as random effect |
| **18-month biomarkers** | 18-month systemic inflammatory biomarkers  18-month enteropathy biomarkers | Infant sex^2^ | Factor in adjusted models |
|  |  | SHINE hub^4^ | Factor in adjusted models |
|  |  | SHINE intervention^4^ | Factor in adjusted models |
|  |  | SHINE cluster^5^ | Included as random effect |
| **Baseline maternal health** | Maternal age | - | No adjustment |
|  | Maternal height | - | No adjustment |
|  | Maternal MUAC | Age^2^, HIV^2^ | Factors in adjusted models |
|  | Maternal HIV status | Age^2^ | Factor in adjusted models |
|  | Maternal  *S. haematobium* status | Age^2^ | Factor in adjusted models |
|  | Maternal systemic inflammation biomarkers | Age^2^, HIV^2^, *S.hamatobium*^2^ | Factors in adjusted models |
|  | Maternal enteropathy biomarkers | Age^2^, HIV^2^, *S.hamatobium*^2^ | Factors in adjusted models |
|  |  | SHINE cluster^5^ | Included as random effect |
| **WASH** | WASH intervention | 18-month stunting status^3^ | Factor in adjusted models |
|  |  | SHINE hub^4^ | Factor in adjusted models |
|  |  | SHINE cluster^5^ | Included as random effect |

^1^Exposure variables were selected from the SHINE dataset (refer to **Supplementary Table 2** for full variable list); variables without a plausible association with immune function (outcome) based on existing literature or within-dataset confounder analyses (i.e. testing for association between outcome and exposure for each model) were not included. Variables with a plausible association but ≥20% missingness, fewer than 10 total events (continuous variables) or events per category (categorical variables) were also not included.

^2^Association with exposure and outcome determined by literature search and associated directed acyclic graph (DAG) for each model.

^3^Association with exposure and outcome determined by within-cohort confounder analyses

^4^As a sub-study of a randomised controlled trial (SHINE), SHINE intervention arm was included in adjustment sets for all models where the exposure variable was influenced by the intervention (i.e. all timepoints after SHINE baseline visit). SHINE hub was also included in adjusted models for post-baseline timepoints to account for whole blood cultures being conducted across separate SHINE hub laboratories (Shurugwi District Hospital and St. Theresa’s Hospital) and because some exposures varied by site (e.g. urogenital schistosomiasis was more prevalent among families recruited in Shurugwi versus St. Theresa’s[1]).

^5^SHINE cluster was the unit of randomisation within SHINE and corresponds to the catchment area of 1-4 Village Health Workers.

**Supplementary Table 2: Comparison of all household, maternal & infant characteristics of the immune function sub-study cohort versus SHINE participants not enrolled in the sub-study**

| **Characteristics** | **Immune function sub-study** | **SHINE cohort not enrolled in sub-study** | **p^1^** |
| --- | --- | --- | --- |
| **Mothers, N^2^** | 111 | 4,264 |  |
| **Infants, N** | 113 | 4,314 |  |
| **SHINE hub^3^ (infant samples):** |  |  |  |
| Shurugwi District Hospital, n/N (%) | 81/113 (71.7%) | 1,404/4,314 (32.5%) |  |
| St. Theresa’s Hospital, n/N (%) | 32/113 (28.3%) | 972/4,314 (22.5%) |  |
| **SHINE intervention arms (infants):** |  |  | <0.001 |
| SOC, n/N (%) | 42/113 (37.2%) | 1016/4314 (23.6%) |  |
| IYCF, n/N (%) | 38/113 (33.6%) | 1021/4314 (23.7%) |  |
| WASH, n/N (%) | 16/113 (14.2%) | 1101/4314 (25.5%) |  |
| IYCF & WASH, n/N (%) | 17/113 (15.0%) | 1176/4314 (27.3%) |  |
| **Household characteristics at enrolment^4^:** |  |  |  |
| Number of Occupants, median (IQR) [n] | 4 (3; 6) [109] | 5 (3; 6) [4,081] | 0.242 |
| Wealth Quintile, n/N (%): |  |  | 0.153 |
| 1 (lowest) | 18/93 (19.4%) | 762/3,934 (19.4%) |  |
| 2 (second) | 19/93 (20.4%) | 784/3,934 (19.9%) |  |
| 3 (middle) | 28/93 (30.1%) | 791/3,934 (20.1%) |  |
| 4 (fourth) | 11/93 (11.8%) | 801/3,934 (20.4%) |  |
| 5 (highest) | 17/93 (18.3%) | 796/3,934 (20.2%) |  |
| Household sanitation: |  |  |  |
| Open defecation, n/N (%) | 44/92 (47.8%) | 1919/3,751 (51.2%) | 0.544 |
| Any latrine, n/N (%) | 34/93 (36.6%) | 1420/3,877 (36.6%) | 0.991 |
| Improved latrine, n/N (%) | 28/93 (30.1%) | 1240/3,872 (32.0%) | 0.733 |
| Improved latrine, well-trodden path, not shared, n/N (%) | 19/88 (21.6%) | 952/3,750 (25.4%) | 0.519 |
| Access to water: |  |  |  |
| Improved source of household drinking water, n/N (%) | 63/93 (67.7%) | 2443/3,893 (62.8%) | 0.348 |
| Treated drinking water, n/N (%) | 6/93 (6.5%) | 491/3,832 (12.8%) | 0.069 |
| One way walk to fetch water, median (IQR) [n], min | 10 (5; 15) [93] | 10 (5; 20) [3,882] | 0.285 |
| Water volume collected per person in last 24 hr, median (IQR) [n], litres | 7 (5; 11) [76] | 7 (5; 12) [3,260] | 0.960 |
| Hygiene: |  |  |  |
| Handwashing station at home, n/N (%) | 12/81 (14.8%) | 332/3,690 (9.0%) | 0.078 |
| Handwashing station with water and rubbing agent, n/N (%) | 0/81 (0.0%) | 26/3,679 (0.7%) | <0.001 |
| Improved floor, n/N (%) | 48/92 (52.2%) | 2110/3,886 (54.3%) | 0.717 |
| Number of chickens, median (IQR) [n] | 6 (2; 10) [95] | 6 (2; 10) [3,914] | 0.792 |
| Livestock observed inside home, n/N (%) | 38/106 (35.9%) | 1,539/4,148 (37.1%) | 0.803 |
| Faeces observed in the yard, n/N (%) | 26/106 (24.5%) | 1,294/4,133 (31.3%) | 0.166 |
| Diet quality and food security: |  |  |  |
| Household meets minimum Diet Diversity Score, n/N (%) | 29/82 (35.4%) | 1,371/3,450 (39.7%) | 0.376 |
| Coping Strategies Index score, median (IQR) [n] | 0 (0; 5) [92] | 1 (0; 4) [3,838] | 0.005 |
| **Maternal characteristics at enrolment^4^:** |  |  |  |
| Age, mean (SD) [n], years | 27 (6) [91] | 26 (7) [3,790] | 0.208 |
| Height, mean (SD) [n], cm | 161 (7) [108] | 160 (9) [4,143] | 0.039 |
| MUAC, mean (SD) [n], cm | 27 (3) [108] | 26 (3) [4,181] | 0.300 |
| Education, years of schooling completed, mean (SD) [n] | 10 (2) [99] | 10 (2) [4,036] | 0.997 |
| Parity, median (IQR) [n] | 2 (1; 3) [94] | 2 (1; 3) [2,953] | 0.477 |
| Married, n/N (%) | 94/99 (95.0%) | 3,811/4,005 (95.2%) | <0.001 |
| Employed, n/N (%) | 6/93 (6.5%) | 343/3,930 (8.7%) | 0.388 |
| Religion, n/N (%): |  |  |  |
| Apostolic | 40/99 (40.4%) | 1,896/4,037 (47.0%) | 0.429 |
| Other Christian | 51/99 (51.5%) | 1,803/4,037 (44.7%) |  |
| Other | 8/99 (8.1%) | 338/4,037 (8.4%) |  |
| *Schistosoma haematobium* ova in urine, n/N (%) | 12/92 (13.0%) | 400/3,732 (10.7%) | 0.524 |
| Anaemic (haemoglobin<12g/dL), n/N (%) | 41/86 (47.7%) | 1,611/3,498 (46.1%) | 0.770 |
| HIV status, n/N (%): |  |  | 0.035 |
| Positive | 18/111 (16.2%) | 639/4,264 (15.0%) |  |
| Negative | 88/111 (79.3%) | 3,559/4,264 (83.5%) |  |
| Unknown | 5/111 (4.5%) | 66/4,264 (1.6%) |  |
| CD4 count, mean (SD) [n], cells/mL | 626 (249) [16] | 482 (218) [555] | 0.003 |
| Antiretroviral therapy (ART) during pregnancy, n/N (%) | 16/16 (100.0%) | 529/559 (94.6%) | <0.001 |
| Prophylactic cotrimoxazole during pregnancy, n/N (%) | 12/13 (92.3%) | 358/532 (67.3%) | 0.095 |
| Systemic inflammation: |  |  |  |
| Plasma CRP, median (IQR) [n], ng/mL | 2748 (1074; 7,169) [62] | 2846 (1274; 6425) [1,669] | 0.124 |
| Plasma sCD14 , median (IQR) [n], pg/mL | 1065818 (837107;1337835) [75] | 1108103  (896255; 1415954) [2,062] | 0.242 |
| Intestinal inflammation: |  |  |  |
| Stool MPO, median (IQR) [n], ng/mL | 800  (800; 1088) [83] | 800  (800; 1059) [2,288] | 0.509 |
| Stool neopterin, median (IQR) [n], nmol/L | 41  (35; 71) [83] | 42  (35; 73) [2,285] | 0.660 |
| **Infant characteristics** |  |  |  |
| **At Birth** |  |  |  |
| Female sex, n/N (%) | 60/113 (53.1%) | 215/4,314 (50.0%) | 0.509 |
| Birthweight , mean (SD) [n], kg | 3.2 (0.4) [111] | 3.1 (0.5) [3,945] | 0.100 |
| Birthweight <2.5 kg, n/N (%) | 6/111 (5.4%) | 349/3,945 (8.9%) | 0.279 |
| Institutional delivery, n/N (%) | 104/108 (96.3%) | 3,525/3,986 (88.4%) | 0.016 |
| Vaginal delivery, n/N (%) | 102/111 (91.9%) | 3,747/4,049 (92.5%) | 0.440 |
| Birth Outcome, n/N (%): |  |  |  |
| Term, AGA | 45/49 (91.8%) | 1,744/2,345 (74.4%) | 0.010 |
| Preterm, AGA | 4/49 (8.2%) | 601/2,345 (25.6%) |  |
| Preterm, SGA | 0/8 (0.0%) | 35/368 (9.5%) | <0.001 |
| **At 18 months** |  |  |  |
| Age at 18-month visit, mean (SD) [n], months | 18.4 (1.6) [113] | 18.7 (1.8) [4,420] | 0.023 |
| Anthropometry: |  |  |  |
| LAZ, mean (SD) [n] | -1.6 (1.1) [113] | -1.6 (1.1) [4,299] | 0.930 |
| WHZ, mean (SD) [n] | 0.02 (1.1) [113] | 0.04 (1.1) [4,282] | 0.791 |
| WAZ, mean (SD) [n] | -0.7 (1.1) [113] | -0.7 (1.0) [4,278] | 0.853 |
| MUACZ, mean (SD) [n] | 0.16 (0.9) [113] | 0.01 (0.9) [4,274] | 0.121 |
| HCZ, mean (SD) [n] | -0.1 (1.1) [113] | -1.6 (1.1) [4,229] | 0.203 |
| Stunted (LAZ<-2) | 44/113 (38.9%) | 1,426/4,299 (33.2%) | 0.225 |
| Anaemic (haemoglobin<10.5g/dL), n/N (%) | 12/113 (10.6%) | 502/4,201 (12.0%) | 0.682 |
| Caregiver-reported symptoms^5^, n/N (%): |  |  |  |
| Any symptom | 11/112 (9.2%) | 914/4,313 (21.2%) | 0.002 |
| Diarrhoea | 2/113 (1.8%) | 397/4,273 (9.3%) | 0.007 |
| Acute respiratory infection | 0/96 (0.0%) | 20/3,114 (0.6%) | - |
| Fever | 5/96 (5.2%) | 203/3,104 (6.5%) | 0.550 |
| Cough | 7/95 (7.4%) | 454/3,110 (14.6%) | 0.023 |
| Pus from ear | 0/95 (0.0%) | 13/3,102 (0.4%) | - |
| Difficulty feeding | 0/95 (0.0%) | 26/3,113 (0.8%) | - |
| Blood in stool | 0/95 (0.0%) | 7/3,059 (0.2%) | - |
| Mucus in stool | 0/95 (0.0%) | 42/3,040 (1.4%) | - |
| Systemic inflammation: |  |  |  |
| Plasma sCD14 , median (IQR) [n], pg/mL | 1387951 (1082782; 1702291) [82] | 1389403 (1119682; 1688879) [1,369] | 0.997 |
| Plasma CRP, median (IQR) [n], ng/L | 1314 (42; 5957) [82] | 725 (116; 3433) [1,369] | 0.598 |
| Growth factors: |  |  |  |
| Plasma IGF-1, median (IQR) [n], ng/mL | 16 (10; 29) [82] | 22 (13; 32) [1,369] | 0.010 |
| Intestinal inflammation: |  |  |  |
| Stool MPO, median (IQR) [n], ng/mL | 1887 (1070; 4118) [72] | 2031 (927; 4123) [1,402] | 0.856 |
| Stool neopterin, median (IQR) [n], nmol/L | 356 (109; 785) [72] | 314 (121; 733) [1,401] | 0.937 |
| Biomarkers of gut damage: |  |  |  |
| Plasma IFABP, median (IQR) [n], pg/mL | 1230 (842; 1659) [82] | 1,157 (805; 1740) [1,369] | 0.470 |
| Stool AAT, median (IQR) [n], ng/mL | 314787 (142167; 588501) [72] | 211691 (112626; 400321) [1,401] | 0.006 |
| HIV status, n/N (%): |  |  |  |
| HUU | 95/113 (84.1%) | 3,664/4,314 (84.9%) | - |
| HEU | 18/113 (16.0%) | 576/4,314 (13.4%) |  |
| HIV positive | 0/113 (0.0%) | 22/4,314 (0.5%) |  |
| HIV unknown | 0/113 (0.0%) | 52/4,314 (1.2%) |  |
| ART initiated for HEU, n/N (%) | 16/16 (100.0%) | 540/554 (97.5%) | <0.001 |
| Cotrimoxazole initiated for HEU, n/N (%) | 12/13 (92.3%) | 370/489 (75.7%) | 0.201 |

SOC – standard of care, IYCF – infant and young child feeding intervention, WASH – household water, sanitation and hygiene intervention; MUAC – mid-to-upper arm circumference; CRP – capsular polysaccharide-reactive protein, sCD14 – soluble CD14 (LPS receptor/TLR4 co-receptor), MPO – myeloperoxidase; ART – antiretroviral therapy (for HIV infection/exposure); SGA – small for gestational age, AGA – appropriate for gestational age; LAZ – length-for-age Z score, WHZ – weight-for-height Z score, WAZ – weight-for-age Z score, MUACZ – MUAC Z score, HCZ – head circumference Z score; IGF-1 – insulin-like growth factor 1, IFABP – intestinal fatty acid binding protein, AAT - α-1-antitrypsin; HUU – HIV-unexposed, uninfected, HEU – HIV-exposed uninfected

**^1^**p-values from general estimating equations (xtgee) for comparing means; Somers' D for comparing medians; and, logit regression for comparing proportions.

^2^The sub-study includes 1 set of twins

^3^SHINE recruited participants at 4 study hubs (Shurugwi District Hospital, St. Theresa’s Hospital, Tongogara clinic and Mvuma clinic); only Shurugwi and St. Theresa’s participants are reported here as these were the only hubs that had laboratory facilities suitable for whole blood culture

^4^Pregnant women were enrolled during pregnancy; in the main SHINE trial, median gestational age at enrolment was 12 (IQR 9–16) weeks[2]. Maternal and household characteristics were captured at the baseline visit after study enrolment.

^5^Children’s symptoms were captured by a 7-day recall questionnaire delivered to their caregivers

**Supplementary Table 3: Censored log-normal (tobit) regression analysis of the relationship between stunting status and unstimulated concentrations of IL-12p70, hepcidin, sCD163 and IFNβ at 18 months of age**

| **Outcome** | **Unadjusted** | | |  | **Adjusted^1^ for SHINE Arm,**  **Hub, Sex & Birthweight** | | |
| --- | --- | --- | --- | --- | --- | --- | --- |
|  | **GMD^1^** | **95%CI** | **p** |  | **Adj. GMD^1^** | **Adj. 95%CI** | **p** |
| **Unstimulated:** |  |  |  |  |  |  |  |
| IL-12p70 | 1.17 | 0.20; 7.03 | 0.860 |  | 0.84 | 0.11; 6.49 | 0.871 |
| Hepcidin^2^ | **3.35** | **1.19; 9.49** | **0.022** |  | **8.5** | **2.83; 25.27** | **<0.001** |
| sCD163 | 1.16 | 1.00; 1.36 | 0.054 |  | 1.16 | 0.98; 1.38 | 0.080 |
| IFNβ | 1.15 | 0.14; 9.77 | 0.894 |  | 2.46 | 0.23; 26.04 | 0.456 |

^1^Geometric mean difference in mediator concentration (pg/mL) between the stunted group (n=44) and the non-stunted group (n=69) estimated from censored log-normal (tobit) regression coefficient; bolded text indicates mediators with evidence for an association with 18-month stunting status (p<0.05).

^2^Unstimulated hepcidin concentrations positively correlated with 18-month plasma hepcidin concentrations (Pearson’s r: 0.45 (0.29, 0.59); p<0.001); there was no evidence that hepcidin concentrations in antigen-stimulated hepcidin levels were higher than those in unstimulated cultures.

**Supplementary Table 4: Censored log-normal (tobit) regression analysis of the relationship between biomarkers of systemic inflammation, enteropathy and anti-bacterial immune function at 18 months of age**

| **Outcome** | | **Unadjusted** | | |  | **Adjusted for SHINE Arm, Hub, Maternal HIV & Sex** | | |
| --- | --- | --- | --- | --- | --- | --- | --- | --- |
|  |  | **GMD^1^** | **95% CI** | **p** |  | **Adj. GMD^1^** | **Adj. 95% CI** | **p** |
| **LPS-specific TNFα^2^** | sCD14^3^ | 1.00 | 1.00; 1.00 | 0.115 |  | 1.00 | 1.00; 1;00 | 0.067 |
|  | CRP^3^ | 1.19 | 0.90; 1.58 | 0.214 |  | 1.13 | 0.87; 1.47 | 0.354 |
|  | MPO^4^ | 0.76 | 0.27; 2.16 | 0.612 |  | 0.61 | 0.23; 1.64 | 0.330 |
|  | Neopterin^4^ | 1.14 | 0.58; 2.23 | 0.708 |  | 1.02 | 0.55; 1.91 | 0.942 |
|  | IFABP^3^ | 0.88 | 0.22; 3.41 | 0.839 |  | 0.92 | 0.25; 3.38 | 0.894 |
|  | AAT^4^ | **0.41** | **0.17; 0.98** | **0.044** |  | **0.36** | **0.16; 0.81** | **0.013** |
| **HKST-specific TNFα^2^** | sCD14^3^ | 1.00 | 1.00; 1.00 | 0.149 |  | 1.00 | 1.00; 1.00 | 0.425 |
|  | CRP^3^ | 1.03 | 0.87; 1.22 | 0.712 |  | 1.01 | 0.89; 1.15 | 0.831 |
|  | MPO^4^ | 1.09 | 0.61; 1.94 | 0.779 |  | 0.86 | 0.55; 1.34 | 0.499 |
|  | Neopterin^4^ | 1.34 | 0.92; 1.95 | 0.128 |  | 1.23 | 0.91; 1.67 | 0.173 |
|  | IFABP^3^ | **0.36** | **0.17; 0.77** | **0.009** |  | **0.44** | **0.24; 0.81** | **0.009** |
|  | AAT^4^ | 0.91 | 0.55; 1.51 | 0.714 |  | 0.85 | 0.56; 1.27 | 0.425 |
| **LPS-specific**  **IL-6^2^** | sCD14^3^ | 1.00 | 1.00; 1.00 | 0.179 |  | 1.00 | 1.00; 1.00 | 0.492 |
|  | CRP^3^ | 1.04 | 0.83; 1.29 | 0.755 |  | 0.97 | 0.81; 1.16 | 0.731 |
|  | MPO^4^ | 0.75 | 0.37; 1.49 | 0.408 |  | 0.59 | 0.34; 1.03 | 0.065 |
|  | Neopterin^4^ | 1.13 | 0.72; 1.78 | 0.584 |  | 0.97 | 0.67; 1.40 | 0.876 |
|  | IFABP^3^ | 0.51 | 0.18; 1.41 | 0.191 |  | 0.62 | 0.26; 1.48 | 0.278 |
|  | AAT^4^ | 0.70 | 0.38; 1.29 | 0.255 |  | 0.72 | 0.43; 1.18 | 0.189 |
| **HKST-specific**  **IL-6^2^** | sCD14^3^ | 1.00 | 1.00; 1.00 | 0.087 |  | 1.00 | 1.00; 1.00 | 0.129 |
|  | CRP^3^ | 1.03 | 0.86; 1.24 | 0.726 |  | 1.02 | 0.89; 1.16 | 0.796 |
|  | MPO^4^ | 1.17 | 0.64; 2.12 | 0.615 |  | 0.87 | 0.55; 1.35 | 0.526 |
|  | Neopterin^4^ | 1.23 | 0.83; 1.83 | 0.308 |  | 1.12 | 0.82; 1.51 | 0.482 |
|  | IFABP^3^ | **0.41** | **0.18; 0.96** | **0.040** |  | 0.54 | 0.28; 1.04 | 0.066 |
|  | AAT^4^ | 1.28 | 0.72; 2.27 | 0.409 |  | 1.10 | 0.71; 1.71 | 0.656 |
| **LPS-specific**  **IL-8^2^** | sCD14^3^ | 1.00 | 1.00; 1.00 | 0.363 |  | 1.00 | 1.00; 1.00 | 0.605 |
|  | CRP^3^ | 0.93 | 0.76; 1.15 | 0.528 |  | 0.88 | 0.73; 1.06 | 0.175 |
|  | MPO^4^ | 0.53 | 0.27; 1.03 | 0.062 |  | **0.44** | **0.24; 0.82** | **0.009** |
|  | Neopterin^4^ | 1.16 | 0.73; 1.84 | 0.526 |  | 1.07 | 0.69; 1.67 | 0.755 |
|  | IFABP^3^ | 0.55 | 0.21; 1.44 | 0.227 |  | 0.64 | 0.25; 1.61 | 0.344 |
|  | AAT^4^ | 0.61 | 0.34; 1.11 | 0.108 |  | 0.60 | 0.34; 1.06 | 0.078 |
| **HKST-specific**  **IL-8^2^** | sCD14^3^ | 1.00 | 1.00; 1.00 | 0.072 |  | 1.00 | 1.00; 1.00 | 0.186 |
|  | CRP^3^ | 1.00 | 0.82; 1.22 | 0.995 |  | 0.98 | 0.83; 1.15 | 0.764 |
|  | MPO^4^ | 1.08 | 0.58; 2.04 | 0.805 |  | 0.85 | 0.50; 1.42 | 0.533 |
|  | Neopterin^4^ | 1.32 | 0.87; 2.01 | 0.191 |  | 1.24 | 0.87;1.76 | 0.241 |
|  | IFABP^3^ | 0.50 | 0.21; 1.20 | 0.122 |  | 0.68 | 0.31; 1.49 | 0.336 |
|  | AAT^4^ | 0.70 | 0.40; 1.22 | 0.203 |  | 0.68 | 0.42; 1.08 | 0.099 |
| **LPS-specific MPO^2^** | sCD14^3^ | 1.00 | 1.00; 1.00 | 0.327 |  | 1.00 | 1.00; 1.00 | 0.568 |
|  | CRP^3^ | 0.91 | 0.65; 1.27 | 0.580 |  | 0.92 | 0.66; 1.28 | 0.625 |
|  | MPO^4^ | 1.72 | 0.56; 5.31 | 0.344 |  | 1.44 | 0.48; 4.31 | 0.516 |
|  | Neopterin^4^ | **2.27** | **1.06; 4.85** | **0.035** |  | **2.48** | **1.19; 5.16** | **0.015** |
|  | IFABP^3^ | 1.40 | 0.26; 7.50 | 0.695 |  | 2.44 | 0.47; 12.84 | 0.291 |
|  | AAT^4^ | 0.48 | 0.17; 1.39 | 0.175 |  | 0.47 | 0.17; 1.27 | 0.135 |
| **HKST-specific MPO^2^** | sCD14^3^ | 1.00 | 1.00; 1.00 | 0.376 |  | 1.00 | 1.00; 1.00 | 0.466 |
|  | CRP^3^ | 0.91 | 0.77; 1.15 | 0.564 |  | 0.97 | 0.80; 1.19 | 0.787 |
|  | MPO^4^ | 1.19 | 0.60; 2.39 | 0.616 |  | 1.09 | 0.56; 2.15 | 0.792 |
|  | Neopterin^4^ | 1.33 | 0.83; 2.13 | 0.235 |  | 1.39 | 0.88; 2.18 | 0.154 |
|  | IFABP^3^ | 0.77 | 0.28; 2.17 | 0.627 |  | 1.02 | 0.37; 2.81 | 0.968 |
|  | AAT^4^ | 0.75 | 0.39; 1.46 | 0.403 |  | 0.77 | 0.42; 1.41 | 0.398 |

^1^Geometric mean difference in mediator concentration (pg/mL) per unit difference in biomarker concentration estimated from censored log-normal (tobit) regression coefficient; bolded text indicates mediators with evidence for an association with the systemic (plasma sCD14, plasma CRP) or intestinal (stool MPO, stool neopterin, stool AAT, plasma IFABP) inflammatory biomarker or plasma Insulin-like growth factor 1 (IGF-1); p<0.05

^2^ΔConcentrations between antigen-stimulated and unstimulated whole blood culture supernatant

^3^Plasma biomarkers n=82

^4^Stool biomarkers n=72

**Supplementary Table 5: Censored log-normal (tobit) regression analysis of the effect of the interaction between WASH and IYCF intervention arms on immune mediator production at 18 months of age**

| **Outcome** | **Unadjusted^1^** | | |
| --- | --- | --- | --- |
|  | **β** | **95%CI** | **p** |
| **Unstimulated^2^:** | |  |  |
| TNFα | -0.74 | -2.50; 1.02 | 0.411 |
| IL-6 | -1.34 | -6.06; 3.37 | 0.576 |
| IL-8 | -0.98 | -2.70; 0,73 | 0.261 |
| MPO | -0.32 | -1.14; 0.51 | 0.448 |
| **Antigen-specific^3^:** | |  |  |
| LPS-specific TNFα | -0.61 | -2.75; 1.52 | 0.573 |
| HKST-specific TNFα | 0.96 | -0.95; 2.86 | 0.324 |
| LPS-specific IL-6 | -0.94 | -3.76; 1.87 | 0.511 |
| HKST-specific IL-6 | 0.39 | -1.62; 2.40 | 0.702 |
| LPS-specific IL-8 | -0.25 | -2.50; 1.79 | 0.748 |
| HKST-specific IL-8 | 0.05 | -2.21; 2.31 | 0.966 |
| LPS-specific MPO | -0.52 | -2.76; 1.73 | 0.652 |
| HKST-specific MPO | 1.18 | -0.92; 3.29 | 0.269 |

^1^β: unadjusted censored log-normal (tobit) regression coefficient; 95%CI: 95% confidence interval; Standard of Care (SOC) arm n=42, IYCF arm n=38, WASH arm n=16, WASH+IYCF arm n=17

^2^Concentrations in unstimulated whole blood culture supernatants

^3^ΔConcentrations between antigen-stimulated and unstimulated whole blood culture supernatants

**Supplementary Table 6: Characteristics with evidence for a difference between WASH and no WASH groups in the immune function sub-study cohort**

| **Characteristics** | **WASH** | **No WASH** | **p^1^** |
| --- | --- | --- | --- |
| **Mothers, N^1^** | 32 | 79 |  |
| **Infants, N** | 33 | 80 |  |
| **SHINE hub:** |  |  |  |
| Shurugwi District Hospital, n/N (%) | 28 | 53 |  |
| St. Theresa’s Hospital, n/N (%) | 5 | 27 |  |
| **SHINE intervention arms:** |  |  |  |
| SOC, n/N (%) | 0/33 (0.0%) | 42/80 (52.5%) |  |
| IYCF, n/N (%) | 0/33 (0.0%) | 38/80 (47.5%) |  |
| WASH, n/N (%) | 16/33 (48.5%) | 0/80 (0.0%) |  |
| IYCF & WASH, n/N (%) | 16/33 (51.5%) | 0/80 (0.0%) |  |
| **Household characteristics at enrolment^2^** |  |  |  |
| Hygiene: |  |  |  |
| Handwashing station at home, n/N (%) | 11/24 (45.8%) | 1/57 (1.8%) | 0.001^2^ |
| **Maternal characteristics at enrolment** |  |  |  |
| Systemic inflammation: |  |  |  |
| CRP, median (IQR) [n], ng/mL | 1421 (721; 4040) [19] | 3488 (1392; 8887) [47] | 0.070^2^ |
| **Infant characteristics** |  |  |  |
| **At 18 months** |  |  |  |
| Anthropometry: |  |  |  |
| LAZ, mean (SD) [n] | -1.9 (1.2) [33] | -1.5 (1.0) [80] | 0.091^2^ |
| Stunted (LAZ<-2) | 17/33 (51.5%) | 27/80 (33.8%) | 0.069 |
| Anaemic (haemoglobin<10.5g/dL), n/N (%)^3^ | 4/33 (12.1%) | 8/80 (10.0%) | 0.738 |
| Caregiver-reported symptoms, n/N (%)^3^: |  |  |  |
| Any symptom | 2/32 (6.3%) | 9/80 (11.3%) | 0.418 |
| Diarrhoea | 1/33 (3.0%) | 1/80 (1.3%) | 0.002^2^ |
| HIV status, n/N (%)^3^: |  |  |  |
| HUU | 29/33 (87.9%) | 66/80 (82.5%) | - |
| HEU | 4/33 (12.1%) | 14/80 (17.5%) |  |
| HIV | 0/33 (0.0%) | 0/80 (0.0%) |  |

^1^All household, maternal and infant characteristics were compared between groups (**Supplementary Table 2** for full variable list); characteristics with p<0.1 for comparisons between arms are shown. General estimating equations (xtgee) were used for comparing means; Somers' D for comparing medians; and, logit regression models for comparing proportions.

^2^Multivariable models to compare unstimulated mediators (**Table 4**; **Supplementary Table 8**) and antigen-specific immune function (**Table 4**) by WASH were adjusted for SHINE hub and stunting status to account for differences in the proportion of stunted children in the WASH vs no WASH arm and evidence that immune function was associated with stunting status (**Figure 3**; **Table 2**); differences between arms by LAZ and CRP were not adjusted for as there was no evidence for a relationship with immune function outcome variables at 18 months of age; household handwashing station at enrolment and diarrhoea were not adjusted for as these variables had <10 events per group.

^3^Sensitivity analyses for the modifying effect of anaemia, HIV-exposure and caregiver-reported symptoms on the relationship between WASH exposure and immune function were not done due to low participant number per group

**Supplementary Table 7: Characteristics with evidence for a difference between IYCF and no IYCF groups in the immune function sub-study cohort**

| **Characteristics** | **IYCF** | **No IYCF** | **p^1^** |
| --- | --- | --- | --- |
| **Mothers, N^1^** | 54 | 57 |  |
| **Infants, N** | 55 | 58 |  |
| **SHINE hub:** |  |  |  |
| Shurugwi District Hospital, n/N (%) | 38 | 43 |  |
| St. Theresa’s Hospital, n/N (%) | 17 | 15 |  |
| **SHINE intervention arms:** |  |  |  |
| SOC, n/N (%) | 0/55 (0.0%) | 42/58 (72.4%) |  |
| IYCF, n/N (%) | 38/55 (69.1%) | 0/58 (0.0%) |  |
| WASH, n/N (%) | 0/55 (0.0%) | 16/58 (27.6%) |  |
| IYCF & WASH, n/N (%) | 17/55 (30.9%) | 0/58 (0.0%) |  |
| **Household characteristics at enrolment^2^** |  |  |  |
| Number of Occupants, median (IQR) [n] | 5 (4; 6) [52] | 4 (3; 5) [57] | 0.075^2^ |
| Wealth Quintile, n/N (%): |  |  | 0.001^2^ |
| 1 (lowest) | 10/44 (22.7%) | 8/49 (16.3%) |  |
| 2 (second) | 6/44 (13.6%) | 13/49 (26.5%) |  |
| 3 (middle) | 19/44 (43.2%) | 9/49 (18.4%) |  |
| 4 (fourth) | 6/44 (13.6%) | 5/49 (10.2%) |  |
| 5 (highest) | 3/44 (6.8%) | 14/49 (28.6%) |  |
| Access to water: |  |  |  |
| One way walk to fetch water, median (IQR) [n], min | 9 (5; 10) [44] | 10 (5; 10) [49] | 0.027^2^ |
| Hygiene: |  |  |  |
| Livestock observed inside home, n/N (%) | 24/52 (46.2%) | 14/54 (25.9%) | 0.031^2^ |
| **Infant characteristics** |  |  |  |
| **At 18 months** |  |  |  |
| Stunted (LAZ<-2) | 22/55 (40.0%) | 22/58 (37.9%) | 0.832^3^ |
| Anaemic (haemoglobin<10.5g/dL), n/N (%)^3^ | 6/55 (10.9%) | 6/58 (10.3%) | 0.925 |
| Caregiver-reported symptoms, n/N (%)^3^: |  |  |  |
| Any symptom | 6/55 (10.9%) | 5/57 (8.8%) | 0.671 |
| HIV status, n/N (%)^3^: |  |  |  |
| HUU | 46/55 (83.6%) | 49/58 (84.5%) | - |
| HEU | 9/55 (16.4%) | 9/58 (15.5%) |  |
| HIV | 0/55 (0.0%) | 0/58 (0.0%) |  |
| Biomarkers of gut damage: |  |  |  |
| Plasma IFABP, median (IQR) [n], pg/mL | 1134 (817; 1483) [38] | 1281 (851; 1738) [44] | 0.022^2^ |

^1^All household, maternal and infant characteristics were compared between groups (**Supplementary Table 2** for full variable list); characteristics with p<0.1 for comparisons between arms are shown. General estimating equations (xtgee) were used for comparing means; Somers' D for comparing medians; and, logit regression models for comparing proportions.

^2^Multivariable models to compare unstimulated mediators (**Table 5**; **Supplementary Table 8**) and antigen-specific immune function (**Table 5**) by IYCF were adjusted for SHINE hub and stunting status; differences between arms by number of household occupants, wealth quintile, one-way walk to fetch water and livestock observed in the home were not adjusted for as there was no evidence for a relationship with immune function variables at 18 months of age. Plasma IFABP was not adjusted for as >20% of participants did not have available data.

^3^Stunting status was included in model adjustment sets due to evidence for an association between stunting and immune function (**Figure 3**; **Table 2**) and because the IYCF intervention reduced stunting prevalence in SHINE[2; 3].

^3^Sensitivity analyses for the modifying effect of anaemia, HIV-exposure and caregiver-reported symptoms on the relationship between IYCF exposure and immune function were not done due to low participant number per group

**Supplementary Table 8: Censored log-normal (tobit) regression analysis of the relationship between the WASH and IYCF interventions and concentrations of IL-12p70, hepcidin, sCD163 and IFNβ in unstimulated culture supernatants at 18 months of age**

| **Outcome** | **Unadjusted** | | | **Adjusted for SHINE hub &**  **18-month stunting status** | | | |
| --- | --- | --- | --- | --- | --- | --- | --- |
|  | **GMD^1^** | **95%CI** | **P** |  | **Adj. GMD^1^** | **Adj. 95%CI** | **p** |
| **Unstimulated:** |  |  |  |  |  |  |  |
| **WASH vs. No WASH^2^** |  |  |  |  |  |  |  |
| IL-12p70 | 0.001 | 0.00; ∞^4^ | 0.999 |  | 0.98 | 0.94; 1.02 | 0.429 |
| Hepcidin | 1.21 | 0.35; 4.18 | 0.762 |  | 1.22 | 0.47; 3.13 | 0.684 |
| sCD163 | 0.98 | 0.83; 1.16 | 0.819 |  | 0.96 | 0.81; 1.14 | 0.639 |
| IFNβ | 0.28 | 0.02; 3.94 | 0.345 |  | 0.99 | 0.71; 1.36 | 0.934 |
| **IYCF vs. No IYCF^3^** |  |  |  |  |  |  |  |
| IL-12p70 | 1.20 | 0.22; 6.55 | 0.834 |  | 1.02 | 0.98; 1.73 | 0.323 |
| Hepcidin | 0.81 | 0.27; 2.41 | 0.703 |  | 0.74 | 0.32; 1.75 | 0.492 |
| sCD163 | 1.00 | 0.85; 1.16 | 0.991 |  | 0.99 | 0.85; 1.15 | 0.927 |
| IFNβ | 0.50 | 0.06; 4.05 | 0.515 |  | 0.89 | 0.66; 1.19 | 0.415 |

^1^Geometric mean difference in mediator concentration (pg/mL) between the intervention group (WASH or IYCF) and the no intervention (reference) group (no WASH or no IYCF) estimated from censored log-normal (tobit) regression coefficient

^2^WASH n=33; no WASH n=80

^3^IYCF n=55; no IYCF n=58

^4^95% CI estimate unreliable as <2% of samples with IL-12p70 concentrations above limit of detection

**SUPPLEMENTARY REFERENCES**

[1] W. Murenjekwa, R. Makasi, R. Ntozini, B. Chasekwa, K. Mutasa, L.H. Moulton, J.M. Tielsch, J.H. Humphrey, L.E. Smith, A.J. Prendergast, and C.D. Bourke, Determinants of Urogenital Schistosomiasis Among Pregnant Women and its Association With Pregnancy Outcomes, Neonatal Deaths, and Child Growth. J Infect Dis 223 (2021) 1433-1444.

[2] J.H. Humphrey, M.N.N. Mbuya, R. Ntozini, L.H. Moulton, R.J. Stoltzfus, N.V. Tavengwa, K. Mutasa, F. Majo, B. Mutasa, G. Mangwadu, C.M. Chasokela, A. Chigumira, B. Chasekwa, L.E. Smith, J.M. Tielsch, A.D. Jones, A.R. Manges, J.A. Maluccio, A.J. Prendergast, J.H. Humphrey, A.D. Jones, A. Manges, G. Mangwadu, J.A. Maluccio, M.N.N. Mbuya, L.H. Moulton, R. Ntozini, A.J. Prendergast, R.J. Stoltzfus, J.M. Tielsch, C. Chasokela, A. Chigumira, W. Heylar, P. Hwena, G. Kembo, F.D. Majo, B. Mutasa, K. Mutasa, P. Rambanepasi, V. Sauramba, N.V. Tavengwa, F. Van Der Keilen, C. Zambezi, D. Chidhanguro, D. Chigodora, J.F. Chipanga, G. Gerema, T. Magara, M. Mandava, T. Mavhudzi, C. Mazhanga, G. Muzaradope, M.T. Mwapaura, S. Phiri, A. Tengende, C. Banda, B. Chasekwa, L. Chidamba, T. Chidawanyika, E. Chikwindi, L.K. Chingaona, C.K. Chiorera, A. Dandadzi, M. Govha, H. Gumbo, K.T. Gwanzura, S. Kasaru, R. Makasi, A.M. Matsika, D. Maunze, E. Mazarura, E. Mpofu, J. Mushonga, T.E. Mushore, T. Muzira, N. Nembaware, S. Nkiwane, P. Nyamwino, S.D. Rukobo, T. Runodamoto, S. Seremwe, P. Simango, J. Tome, B. Tsenesa, U. Amadu, B. Bangira, D. Chiveza, P. Hove, H.A. Jombe, D. Kujenga, L. Madhuyu, P.M. Makoni, N. Maramba, B. Maregere, E. Marumani, E. Masakadze, P. Mazula, C. Munyanyi, G. Musanhu, R.C. Mushanawani, et al., Independent and combined effects of improved water, sanitation, and hygiene, and improved complementary feeding, on child stunting and anaemia in rural Zimbabwe: a cluster-randomised trial. The Lancet Global Health 7 (2019) e132-e147.

[3] A.J. Prendergast, B. Chasekwa, C. Evans, K. Mutasa, M.N.N. Mbuya, R.J. Stoltzfus, L.E. Smith, F.D. Majo, N.V. Tavengwa, B. Mutasa, G.T. Mangwadu, C.M. Chasokela, A. Chigumira, L.H. Moulton, R. Ntozini, and J.H. Humphrey, Independent and combined effects of improved water, sanitation, and hygiene, and improved complementary feeding, on stunting and anaemia among HIV-exposed children in rural Zimbabwe: a cluster-randomised controlled trial. The Lancet Child & Adolescent Health 3 (2019) 77-90.
